# Dynamic Modularity of Host Protein Interaction Networks in *Salmonella* Typhi Infection

**DOI:** 10.1371/journal.pone.0104911

**Published:** 2014-08-21

**Authors:** Paltu Kumar Dhal, Ranjan Kumar Barman, Sudipto Saha, Santasabuj Das

**Affiliations:** 1 Biomedical Informatics Centre, National Institute of Cholera and Enteric Diseases, Kolkata, West Bengal, India; 2 Division of Clinical Medicine, National Institute of Cholera and Enteric Diseases, Kolkata, West Bengal, India; 3 Bioinformatics Centre, Bose Institute, Kolkata, West Bengal, India; Federal University of São Paulo, Brazil

## Abstract

**Background:**

*Salmonella* Typhi is a human-restricted pathogen, which causes typhoid fever and remains a global health problem in the developing countries. Although previously reported host expression datasets had identified putative biomarkers and therapeutic targets of typhoid fever, the underlying molecular mechanism of pathogenesis remains incompletely understood.

**Methods:**

We used five gene expression datasets of human peripheral blood from patients suffering from *S.* Typhi or other bacteremic infections or non-infectious disease like leukemia. The expression datasets were merged into human protein interaction network (PIN) and the expression correlation between the hubs and their interacting proteins was measured by calculating Pearson Correlation Coefficient (PCC) values. The differences in the average PCC for each hub between the disease states and their respective controls were calculated for studied datasets. The individual hubs and their interactors with expression, PCC and average PCC values were treated as dynamic subnetworks. The hubs that showed unique trends of alterations specific to *S.* Typhi infection were identified.

**Results:**

We identified *S.* Typhi infection-specific dynamic subnetworks of the host, which involve 81 hubs and 1343 interactions. The major enriched GO biological process terms in the identified subnetworks were regulation of apoptosis and biological adhesions, while the enriched pathways include cytokine signalling in the immune system and downstream TCR signalling. The dynamic nature of the hubs CCR1, IRS2 and PRKCA with their interactors was studied in detail. The difference in the dynamics of the subnetworks specific to *S. Typhi* infection suggests a potential molecular model of typhoid fever.

**Conclusions:**

Hubs and their interactors of the *S. Typhi* infection-specific dynamic subnetworks carrying distinct PCC values compared with the non-typhoid and other disease conditions reveal new insight into the pathogenesis of *S. Typhi*.

## Introduction

The World Health Organization (WHO) recognizes infection by Gram-negative bacterium *Salmonella enterica* serovar Typhi as a global health problem. The bacterium generally causes an acute febrile illness known as enteric fever, while in some individuals, a chronic carrier state is established that may contribute to the development of adenocarcinoma of the gallbladder [1]. Globally, 22 million typhoid fever cases occur annually, accounting for approximately 6, 00,000 deaths with the highest concentration in Asia, especially in the Indian subcontinent [2]. Mechanism behind the pathogenesis of *Salmonella* Typhi is poorly understood and clinical diagnosis is often unreliable due to overlapping symptoms and signs with other febrile illnesses [3]. Incorrect diagnosis and subsequent use of broad spectrum antibiotics may lead to the rise of multi-drug resistant strains [4]. So long as the understanding of the pathogenesis of typhoid fever remains incomplete, improvement of diagnosis, treatment and vaccine strategies will be delayed.

A host of distinct gene expression profiles are available in the public domain for *Salmonella* Typhi as well as the host during infection [5], [6], [7], [8], [9], [10], [11], [12]. All these data may help to identify enriched gene clusters, which will represent novel pathways that may be targeted to improve diagnostic, prognostic, therapeutic and next-generation vaccine strategies for typhoid fever. A major problem with the expression-based classification is the cellular differences within tissues and genetic variations among the patients suffering from different diseases including typhoid fever, which may weaken the discriminative power of the individual genes [15]. Therefore, unbiased utilization of the data is crucial for the identification of disease mechanisms.

Protein-protein interaction (PPI) network reveals many important aspects of cellular organization and functions. Thus, protein-protein interaction network (PIN) provides a global picture of cellular mechanisms [13]. To better evaluate the roles they play in complex diseases, genes need to be investigated in the PIN where they are involved [14], [15]. Few studies have so far been conducted where integration of gene expression profiles has revealed the dynamics of protein interaction networks and resulted in the identification of condition- or location-specific features of the interactome [16], [17]. Examples of such studies include dynamic modular structure of the human protein interaction network with aging [17] and context-specific or constitutive human protein interaction network in cancer [18]. Studies have also been done with human dilated cardiomyopathy (DCM) to efficiently identify the potential novel DCM signature genes and drug targets [19], [20]. However, cellular mechanisms behind the pathogenesis of infectious diseases, typhoid fever in particular, need to be further explored using similar methods.

Here, a systematic approach was made to develop a network-based analysis by integrating human peripheral blood gene expression profiles during infections with *Salmonella* Typhi, other non-typhoidal *Salmonella*, *E.coli*, *Streptococcus pneumoniae* and acute myeloid leukemia (AML) to human PIN network to discover *Salmonella* Typhi infection-specific subnetworks. Efforts were made to understand the processes of rewiring of the protein-protein interaction network in terms of the co-expression level of the proteins during *Salmonella* Typhi infection. We found changes in the dynamic modularity associated with *Salmonella* Typhi infection that may provide the prognostic markers of typhoid fever. Finally, enriched GO biological processes and biological pathways represented by the subnetworks were also identified. To the best of our knowledge, this is the first report where the pathogenesis of typhoid fever was investigated by the integration of host expression datasets in human PIN and *S.* Typhi infection specific subnetworks were identified followed by characterization of their dynamic nature.

## Materials and Methods

### 2.1 Protein-protein interaction network and gene expression data analysis

The binary protein-protein interaction dataset was downloaded from Human Protein Reference Database (HPRD) [21] (Release 9). This dataset contains the largest connected components currently available and comprises of 9,617 proteins and 39,240 interactions. A parental network named HPRD protein-protein interaction network (PIN) was constructed with these proteins and visualized using Cytoscape 3.0.1 [22].

All the gene expression datasets were downloaded from the Gene Expression Omnibus (GEO) database [23] with the accession number GSE28658 [3], GSE43838 [24], GSE40586 (unpublished) and GDS3057 [25]. All those microarray datasets were already normalized and submitted to the databases by the respective authors. Details of the datasets were described in Table 1. For each dataset, gene expression profiles were processed as previously described [28].

**Table 1:** GEO series (GSE) datasets used in this study.

GEO accession no	No of Sample	Description	Abbreviation	References
GSE28658	3×2	Peripheral blood sample of *Salmonella* Typhi-infected host in acute and convalescent phase.	STA Vs STC	[3]
GSE28658	3×2	Peripheral blood sample of bacteremic (infection *Acinetobacter*, *Klebsiella*, and non-typhoidal *Salmonella*) infected host in acute and convalescent phase	BMA Vs BMC	[3]
GSE43838	6	Blood sample of patients after and before 24 h *Escherichia coli* 83972 incubation.	ECI Vs ECC	[24]
GSE40586	16	Peripheral blood sample of the patients infected and uninfected with *Streptococcus pneumoniae*.	SPI Vs SPC	Unpublished
GDS3057	12	Leukemic blasts from acute myeloid leukemia (AML) patients with normal hematopoietic cells	MLDVsNHC	[25]

### 2.2 Data integration to determine the Pearson Correlation Coefficient (PCC) of co-expression in the interaction networks

The downloaded expression data were formatted uniformly and multiple gene expression spots for a particular gene were averaged for integrating into the static protein-protein interaction network (PIN). Nodes with a degree (k) cut-off greater than or equal to 5 were used for further consideration and named as hubs, which represent the highest 15% of the degree distribution. Correlation of gene expression profiles between the hubs and their interacting proteins (nodes) in the PIN were measured by calculating the PCC between each pair of interacting proteins *i.e.* between the hub and each of its interacting partners (nodes). PCC of paired genes (X and Y), which encode interacting proteins in the PIN, is defined as:
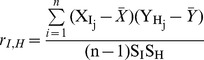
where 
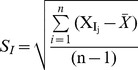
 and 
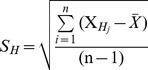
 where, r = Pearson Correlation Coefficient; 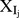 = expression data for interactor I of hub H of individual dataset j = 1, 2, 3…n; 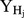 = expression data for hub H of individual dataset j = 1, 2, 3…n; n = total number of values. I is an interactor of hub H, j denotes the expression data for the hub or interactor in each of the n samples and the summation is over all datasets (j = 1, 2, 3…n). S_I_S_H_ is the product of the standard deviations of the expression data for the hub and the interactors. The average PCC of co-expression for each interactor and the hub was assessed using a previously-described algorithm [16], [18], [26]. The average Pearson Correlation Coefficient (Avg. PCC) for all the interactors of hub H was defined as: Avg. PCC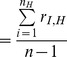, where n is the number of interaction of a particular hub. The “Hmisc” package of R (V.3.0.1) was used to estimate PCC and Avg. PCC values [45].

### 2.3 Identification of *Salmonella* Typhi infection-specific subnetwork

The common hubs that represented all the disease conditions in our study were identified and enlisted (Table S1). Avg. PCC of the common hubs was measured for each of the five diseases and compared with the respective controls. Subsequently, differences between the perturbances and their controls were calculated. The hubs that showed *Salmonella* Typhi infection-specific unique trends of alteration in Avg. PCC were identified and termed as *Salmonella* Typhi infection-specific hubs. All the interactions involving each of these hubs and their PCC values were also documented (Table S2). Larger absolute values of PCC indicate higher correlation between the evaluated gene pairs. A network was constructed using *Salmonella* Typhi infection-specific hubs and their interactors and named as *Salmonella* Typhi infection-specific subnetwork.

### 2.4 Functional group annotation

Biological process GO terms that correspond to the identified subnetworks and enrichment of biological processes in the individual hubs with their interactors were separately analysed using functional annotation clustering (GOTERM_BP_ALL) with medium classification stringency (default settings) of The Database for Annotation, Visualization and Integrated Discovery (DAVID) v6.7 [27], [28] and ClueGO plugin [29], [30] of Cytoscape. To determine the statistical significance of enrichment of the identified subnetworks, two-sided (Enrichment/Depletion) test based on the hypergeometric distribution was used.

### 2.5 Topological network analysis

Characteristic path length (CPL) of the co-expressed networks was calculated as described below. In order to examine whether factors, such as interaction degrees (or ‘betweenness’) have any impact on the network topology of *Salmonella* Typhi infection-specific co-expressed subnetworks, we ordered all the genes in one list according to increasing interaction degrees. Genes were removed starting with the first gene having the lowest degree until the last gene on the list and CPL was calculated as described previously [17], [31], [32].

## Result

### 3.1 Identification of the *S.* Typhi infection-specific dynamic subnetworks

**Figure 1:**
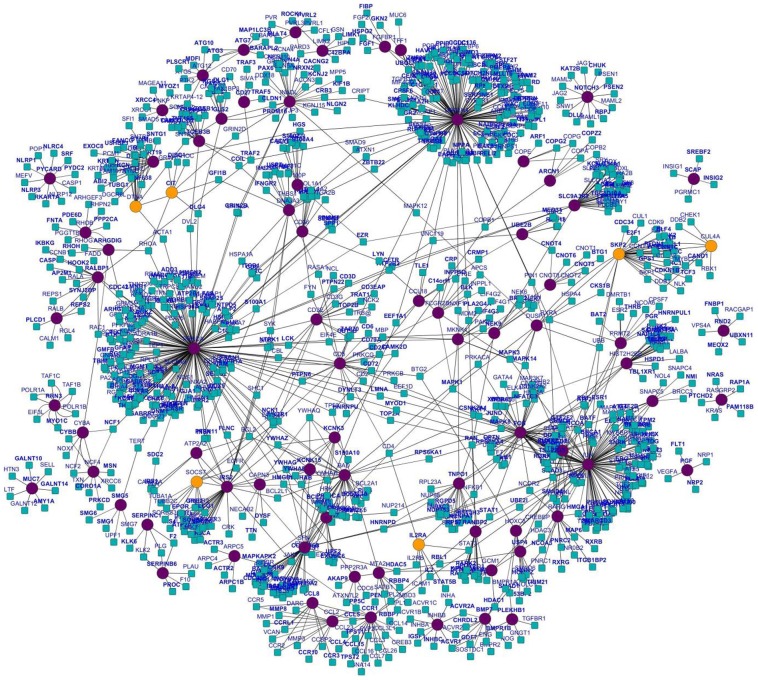
*Salmonella* Typhi infection specific dynamic subnetworks. Co-expression merged with protein interaction networks, specifically altered during acute phase of *S.* Typhi infection. The round and diamond shaped nodes represent the hubs and their interacting partners, respectively. Out of 81, 6 yellow coloured hubs (IL2RA, SKP2, CIT, DTNA, CUL4A and SOCS7) represent increased Avg. PCC, while reaming 75 violet coloured (∼93%) hubs had decreased Avg. PCC during acute phase of *S.* Typhi infection.

Condition-specific subnetwork model allows us to discover the dynamic nature of the network with respect to different infections [33]. The human protein-protein interaction network (PIN) from HPRD consists of 39,240 interactions, in which 9,617 proteins are connected into circuits of protein-protein interactions. The hub proteins with many (≥5) interacting partners in the human PIN were identified, which included 4072 hubs leading to 28811 interactions as shown in Table 2. Next, peripheral blood gene expression data of the five previously-mentioned patients with their respective controls were integrated with the human PIN and the extent of co-expression of a particular hub and its interacting partners was quantified. This identified 3025 common hubs having 17261 interactions present in all the expression datasets (Table 2 and Table S1). Average PCC was computed for all the interactions in which a particular hub participates to calculate the average co-expression score (Table S1). Next, we looked for significant differences in the Avg. PCC of all the common hub proteins between the patients and their respective controls. For example, Avg. PCC of the hub FOS is lower during acute phase of *Salmonella* Typhi infection (0.204) compared to the convalescent phase (0.618). But, this trend of change i.e. decreased Avg. PCC during infection was not found in other conditions studied here, such as other bacterial infections or AML. For example, in case of bacteremic infections, Avg. PCC of FOS showed higher values during the acute phase (0.393) compared to the convalescent phase of infection (−0.298). This analysis helped us to identify the 81 hubs that showed unique trends of changes in *S.* Typhi-infected patients (Table 2). These unique 81 hubs with 1343 interactions were used to construct a network named as *S.* Typhi infection-specific subnetwork (Figure 1). In this study, we did not try to identify hubs, which showed significantly up- or down-regulated expression; instead efforts were made to detect hubs showing unique trends of Avg. PCC alteration or having uniquely-altered PCC with respect to *S.* Typhi infection. High co-expression level (as measured by Avg. PCC) indicates more stable binding between the hub and its interactors, whereas relatively lower co-expression level indicates more dynamic binding [16], [34]. The above 81 unique hubs are preferentially associated with dynamic binding with a bias for decreasing Avg. PCC (Figure 1) with respect to the control. Our data indicated that out of 81 hubs, six (∼7%) showed higher Avg. PCC in the acute phase of *S.* Typhi infection and reaming 75 (∼93%) hubs showed increasing Avg. PCC during the convalescent phase of *S.* Typhi infection.

**Table 2:** Characteristics of protein-protein interaction network used in this study.

Characteristics	No. of hubs	Interactions		Clustering Coefficient	Network Density
		Nodes	Edges		
Protein-protein interaction network from HPRD		9617	39240	0.102	0.001
Protein-protein interaction network from HPRD with degree K≥5	4071	8850	28811	0.136	0.003
Common hubs having expression profile across five GEO studies	3025	8120	17261	0.133	0.004
Specific hubs for *Salmonella* Typhi infection	81	1091	1343	0.034	0.002

### 3.2 Functional annotation of the *S.* Typhi infection-specific dynamic subnetworks

The *Salmonella* Typhi infection-specific subnetworks were functionally annotated and subjected to in-depth analysis. The hubs and their interacting partners in the *Salmonella* Typhi-specific subnetworks were individually tested (each hubs and their interacting partners were considered independently) to find out whether they represent any enriched GO biological processes and are part of functionally important KEGG/or REACTOME pathways. The GO BP terms that represent the highest number of genes with FDR (False Discovery Rate) ≤0.05 were enlisted (Table S3) and used for further analysis. Among the major enriched GO BP, regulation of apoptosis (GO id: 0042981), biological adhesion (GO id: 0022610), cell surface receptor-linked signal transduction (GO id: 0007166) and actin cytoskeleton organization (GO id: 0030036) were observed (Table 3 and Table S3). Significantly enriched pathway terms (P_val_≤0.05) from both KEGG and REACTOME databases were further analysed. The enriched pathways include cytokine signalling in the immune system, downstream TCR signalling, hemostasis, cross-presentation of particulate exogenous antigens (phagosomes) and chemokine receptors bind chemokines etc (Table 4 and Table S4). Each of the significantly enriched pathways (P_val_≤0.05) was separately searched in the PubMed with the subject “pathway term and *Salmonella*/*Salmonella* Typhi” for its relevance, if any in the human host during *Salmonella* Typhi as well as other *Salmonella* spp infection and number of hits were counted and sorted in descending order (Table S5 and Table S6). As indicated in table 3 and 4, hubs ARHGDIG, CCL2, CCR1, CD3E, CYBA, DUSP1, FCGR2B, FOS, IRS2, JUN, NCF4, NOTCH3, PRKCA and PYCARD with their respective interacting partners are associated with more than one pathways. This means that the above-mentioned hubs and their interacting partners bear strong relevance to *S.* Typhi infection. Two such hubs, CCR1 chemokine (C-C motif) receptor and IRS2 (insulin receptor substrate 2) with their interacting partners also showed dynamic nature during *S.* Typhi infection.

**Table 3:** Significantly enriched gene ontology biological process term analysis of 8 hubs and their interactors using DAVID database.

Hub	Interactors	GO Term	FDR
CCR1	CCL3, CCL4, CCL5, CCR1, PLP2, CREB3, JAK1, STAT1, STAT3, CCL26, CCL2, CCL7, CCL14, CCL15, CCL16, CCL3L1, CCL8, CCL23, GNA14, TPST1, TPST2	GO:0006935∼chemotaxis	1.82×10^−18^
CD3E	CD3E,CD3EAP,PIK3R1,ZAP70,CD3D,TRB@,SYK,SHC1,NCK2,TOP2B,CD79B,NCL,UNC119,NCK1,TRAT1,CD3G,PTPN22,LCK	GO:0007166∼cell surface receptor linked signal transduction	1.9×10^−04^
DUSP1	DUSP1,MAPK14,HSPA4,SKP2,CKS1B,MAPK1,UBB,MAPK8,MAPK3,MAPK12	GO:0007265∼Ras protein signal transduction	0.049
FCGR2B	FCGR2B,PTPN6,INPPL1,LY6E,CRP,C14orf1,INPP5D,APCS,LYN,BLK,MAPK1,MAPK3	NA	NA
IRS2	IRS2,PIK3R1,PLCG1,PTPN11,TYK2,ATP2A1,BCL2L1,JAK3,SHC1,UBTF,PTPN6,PIK3CD,PIK3R2,SOCS1,PIK3R3,SOCS7,NTRK1,EPOR,IGF1R,IL4R,JAK1,PTPRF,GRB2,YWHAZ,YWHAE,SOCS6,YWHAG,SOCS3,JAK2,ATP2A2,MPL,CRK,PIK3CA,FES,YWHAB,INSR	GO:0006793∼phosphorus metabolic process	7.66×10^−05^
NOTCH3	NOTCH3,PSEN2,MAML2,MAML3,CHUK,RBPJ,JAG1,JAG2,SNW1,MAML1,DLL1,KAT2B,PSEN1	GO:0007219∼Notch signaling pathway	2.72×10^−17^
PRKCA	KIT, OPRD1, RHOA, SPP1, PAM, PFKFB2, KCNE1, PTPN11, PRKCA, RRAD, RHO, DDX5, SRC, TP53, VCL, VTN, NCF1, GJB1, RPL10, ITGB2, PTPN12, SNAP25, NFATC1, DLX3, CASR, GPM6A, DGKZ, ADD3, GMFB, PA2G4, NRGN, DNM1, PLD1, HAND1, CFTR, CYTH2, RGS7, SNAP23, OCLN, STXBP1, SPAG1, PEA15, GRM1, RGS19, CD163, TRPV6, GSK3A, PLCB1, HSPB8, PEBP1, CHAT, MARCKS, PRKG1, PTGIR, EDF1, ADAP1, TNNI3, HABP4, GABRR1, TRPC3, PPP1R14A, THOC5, GRIA2, HMGB1, RARA, SDC4, TNNT2, ATP2B1, ADRBK1, PTPN6, SLC6A9, KCNQ2, GRM5, F11R, CDC42, ATP2B2, CBL, SHC1, DGKD, ACTA1, GJA1, DVL2, CREM, CORO1B, EZR, TOP2A, FLNC, RALBP1, EEF1D, EGFR, EIF4E, EWSR1, SLC1A1, ITGB1, GABRG2, GFAP, GRIA4, GRIA1, GRIN2B, GRIN2A, GFPT1, GNA15, HES1, HSPA1A, SDC2, HLA-A, CD9, ADRA1B, ITPKA, ITPKB, INSR, KRT18, LMNA, LMNB1, BCL2, ANXA2, CD5, LCK, MGMT, MBP, MYOD1, NOS1, APLP2, HMGN2, HMGN1, RAF1, GNB2L1, PRKCZ, SCTR, SEMG1, SEMG2, TERT, XK, SYK, TIAM1, AVPR1A, RGS2, MYLK, C1QBP, YWHAZ, BTG2, TEP1, ARHGEF1, OGG1, RAC1, PLD2, HAND2, CISH, FSCN1, DLG4, GNA12, AKAP5, AKAP12, YWHAG, POLB, ADCY5, SDPR, KCNE4, AFAP1, TRIM29, PDLIM7, STXBP3, GABRR2, BTK, ANXA7, FAS, ITGB4, PICK1, PPARA, FLNA, NUMB, HSP90AA1, ENTPD5	GO:0044093∼positive regulation of molecular function, GO:0043549∼regulation of kinase activity, GO:0043067∼regulation of programmed cell death	6.77×10^06^
PYCARD	PYCARD,MEFV,SRF,NLRP3,NLRC4,CASP1,NLRP12,PRKAR1A,NLRP1,PYDC2,POP1	GO:0006919∼activation of caspase activity	3.71×10^−05^

**Table 4:** Significantly enriched biological pathway (P value ≤0.05) analysis of 8 hubs and their interactors by the CluGO plug-in (using both KEGG_24.05.2012 and REACTOME_10.07.2012) of Cytoscape_v3.0.1.

Hub	Interactors	REACTOM		KEGG	
		Pathways	P_Value_	Pathways	P_Value_
CCR1	CCL3, CCL4, CCL5, CCR1, PLP2, CREB3, JAK1, STAT1, STAT3, CCL26, CCL2, CCL7, CCL14, CCL15, CCL16, CCL3L1, CCL8, CCL23, GNA14, TPST1, TPST2	Peptide ligand-binding receptors, Chemokine receptors bind chemokines, Regulation of IFNG signalling and Interleukin-6 signaling	2.89×10^−10^ to 1.85×10^−06^	Cytokine-cytokine receptor interaction and Chemokine signaling pathway	7.97×10^−15^ and 7.40×10^−21^
CD3E	CD3E,CD3EAP,PIK3R1,ZAP70,CD3D,TRB@,SYK,SHC1,NCK2,TOP2B,CD79B,NCL,UNC119,NCK1,TRAT1,CD3G,PTPN22,LCK	TCR signalling, Downstream TCR signalling and Generation of second messenger molecules	2.14×10^−14^ to 6.89×10^−11^	ErbB signaling pathway, T cell receptor signaling pathway and Primary immunodeficiency	1.62×10^−05^ to 5.05×10^−07^
DUSP1	DUSP1,MAPK14,HSPA4,SKP2,CKS1B,MAPK1,UBB,MAPK8,MAPK3,MAPK12	Toll-like receptor signalling pathway, Fc epsilon RI signaling pathway and Salmonella infection	2.52×10^−07^ to 1.07×10^−07^	Toll Like Receptor 3 (TLR3) Cascade and MyD88:Mal cascade initiated on plasma membrane	8.30×10^−07^ and 1.16×10^−06^
FCGR2B	FCGR2B,PTPN6,INPPL1,LY6E,CRP,C14orf1,INPP5D,APCS,LYN,BLK,MAPK1,MAPK3	Signaling by Interleukins, Signaling by SCF-KIT and Growth hormone receptor signaling	2.11×10^−07^ to 1.94×10^−08^	B cell receptor signaling pathway and Fc gamma R-mediated phagocytosis	3.68×10^−14^ and 8.46×10^−11^
IRS2	IRS2,PIK3R1,PLCG1,PTPN11,TYK2,ATP2A1,BCL2L1,JAK3,SHC1,UBTF,PTPN6,PIK3CD,PIK3R2,SOCS1,PIK3R3,SOCS7,NTRK1,EPOR,IGF1R,IL4R,JAK1,PTPRF,GRB2,YWHAZ,YWHAE,SOCS6,YWHAG,SOCS3,JAK2,ATP2A2,MPL,CRK,PIK3CA,FES,YWHAB,INSR	Cytokine Signaling in Immune system, Signaling by Interleukins and Interleukin-3, 5 and GM-CSF signaling	3.97×10^−20^ to 4.15×10^−23^	Jak-STAT signaling pathway, Neurotrophin signaling pathway, Insulin signaling pathway and Epstein-Barr virus infection	1.48×10^−22^ to 1.63×10^−11^
NOTCH3	NOTCH3,PSEN2,MAML2,MAML3,CHUK,RBPJ,JAG1,JAG2,SNW1,MAML1,DLL1,KAT2B,PSEN1	Signaling by NOTCH and Regulated proteolysis of p75NTR	6.24×10^−21^ and 2.10×10^−04^	Notch signaling pathway	9.62×10^−26^
PRKCA	KIT, OPRD1, RHOA, SPP1, PAM, PFKFB2, KCNE1, PTPN11, PRKCA, RRAD, RHO, DDX5, SRC, TP53, VCL, VTN, NCF1, GJB1, RPL10, ITGB2, PTPN12, SNAP25, NFATC1, DLX3, CASR, GPM6A, DGKZ, ADD3, GMFB, PA2G4, NRGN, DNM1, PLD1, HAND1, CFTR, CYTH2, RGS7, SNAP23, OCLN, STXBP1, SPAG1, PEA15, GRM1, RGS19, CD163, TRPV6, GSK3A, PLCB1, HSPB8, PEBP1, CHAT, MARCKS, PRKG1, PTGIR, EDF1, ADAP1, TNNI3, HABP4, GABRR1, TRPC3, PPP1R14A, THOC5, GRIA2, HMGB1, RARA, SDC4, TNNT2, ATP2B1, ADRBK1, PTPN6, SLC6A9, KCNQ2, GRM5, F11R, CDC42, ATP2B2, CBL, SHC1, DGKD, ACTA1, GJA1, DVL2, CREM, CORO1B, EZR, TOP2A, FLNC, RALBP1, EEF1D, EGFR, EIF4E, EWSR1, SLC1A1, ITGB1, GABRG2, GFAP, GRIA4, GRIA1, GRIN2B, GRIN2A, GFPT1, GNA15, HES1, HSPA1A, SDC2, HLA-A, CD9, ADRA1B, ITPKA, ITPKB, INSR, KRT18, LMNA, LMNB1, BCL2, ANXA2, CD5, LCK, MGMT, MBP, MYOD1, NOS1, APLP2, HMGN2, HMGN1, RAF1, GNB2L1, PRKCZ, SCTR, SEMG1, SEMG2, TERT, XK, SYK, TIAM1, AVPR1A, RGS2, MYLK, C1QBP, YWHAZ, BTG2, TEP1, ARHGEF1, OGG1, RAC1, PLD2, HAND2, CISH, FSCN1, DLG4, GNA12, AKAP5, AKAP12, YWHAG, POLB, ADCY5, SDPR, KCNE4, AFAP1, TRIM29, PDLIM7, STXBP3, GABRR2, BTK, ANXA7, FAS, ITGB4, PICK1, PPARA, FLNA, NUMB, HSP90AA1, ENTPD5	Hemostasis, Platelet activation, signaling and aggregation, Transmission across Chemical Synapses and Gastrin-CREB signalling pathway via PKC and MAPK	3.08×10^−11^ to 3.33×10^−05^	Focal adhesion, Calcium signaling pathway, Glutamatergic synapse, Regulation of actin cytoskeleton, Chemokine signaling pathway, Endocytosis, Bacterial invasion of epithelial cells and Natural killer cell mediated cytotoxicity	1.68×10^−07^ to 1.07×10^−05^
PYCARD	PYCARD,MEFV,SRF,NLRP3,NLRC4,CASP1,NLRP12,PRKAR1A,NLRP1,PYDC2,POP1	Inflammasomes and Nucleotide-binding domain, leucine rich repeat containing receptor (NLR) signaling pathways	2.07×10^−15^ and 1.37×10^−12^	NOD-like receptor signaling pathway	4.12×10^−11^

### 3.3 Architecture of the *Salmonella* Typhi infection-specific dynamic subnetworks

To evaluate the importance of the 81 unique hubs to the network stability, a topological measure of network connectivity was adopted in the *S.* Typhi-infected uniquely-altered co-expressed network. We used an established test for network structure stability called the change of characteristic path length (CPL) [35], [36]. Attacking the first 71 out of 81unique hubs i.e., the hubs having degree ≤18, did not significantly change the CPL (Figure S1 and Table S7). Attack of the remaining 12 hubs *i.e.*, CCR1, CD5, INADL, SKP2, SLC9A3R2, BAD, IRS2, SFN, FOS, JUN, UBQLN4 and PRKCA (k = 21 to 174) showed a strong antagonistic effect and removing them led to a rapid decrease of the CPL of the network. It is known that the peripheral nodes are relatively less essential for the stability of a network than the centrally located nodes and most of the genes used in this study are peripherally located in the corresponding network.

### 3.4 Identification of the key dynamic interactors in the subnetworks specific to *Salmonella* Typhi infection

**Figure 2:**
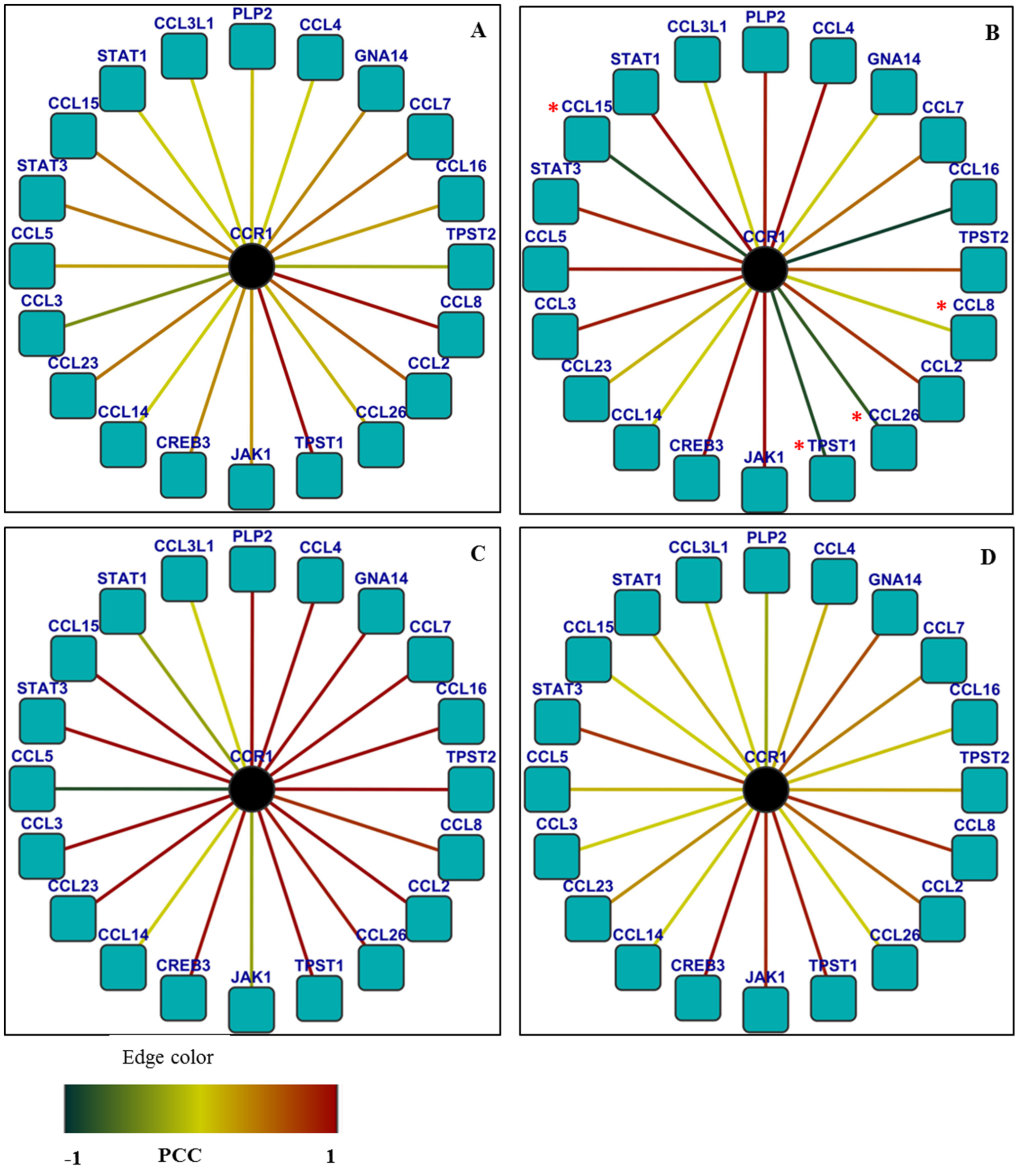
Network of the interacting partners of CCR1 representing the differences in dynamic network properties during *S.* Typhi infection. The edges were labelled with respective PCC values of individual interactors during different perturbances. The four conditions mentioned are A. Control (normal host cell), B. *S.* Typhi infection, C. other bacteremic (non-typhoid Salmonella, *Klebsiella* spp and *Acinetobacter* spp) infections and D. Leukemia. Hub CCR1 and four interactors (e.g, CCL15, CCL26, CCL8 and TPST1) showed unique co-expression patterns (lower PCC values corresponding to correlated expression between protein pairs) specific to *S.* Typhi infection.

**Figure 3:**
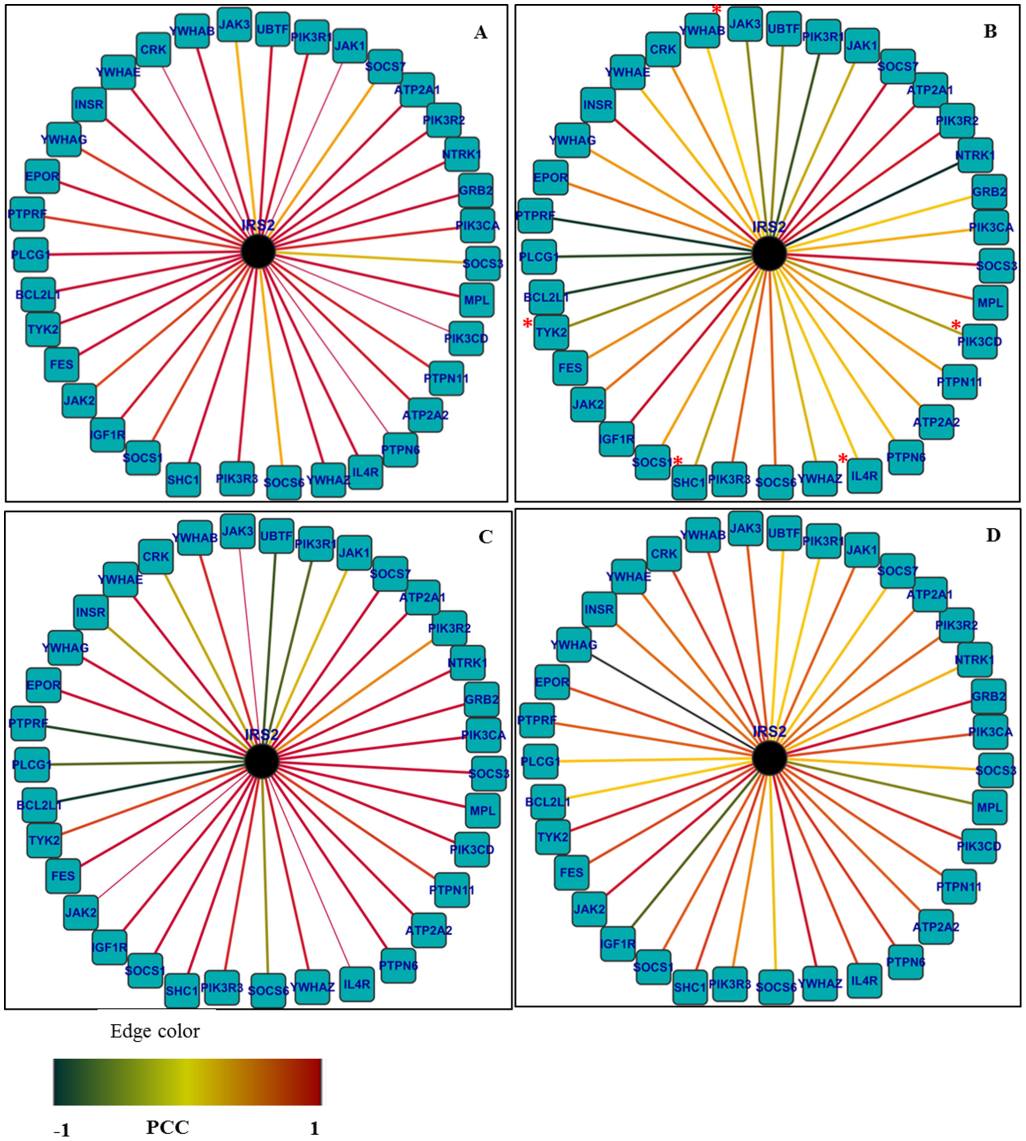
Network of the interacting partners of IRS2 representing differences in dynamic network properties in *S.* Typhi infection. The edges were labelled with respective PCC values of individual interactors during different perturbances. The four mentioned conditions are A. Control (normal host cell), B. *S.* Typhi infection, C. other bacteremic (non-typhoid Salmonella, *Klebsiella* spp and *Acinetobacter* spp) infections, D. Leukaemia. Hub IRS2 and five interactors (e.g, IL4R, JAK3, PIK3CD, SHC1 and TYK2) showed unique co-expression pattern (lower PCC values corresponding to correlated expression between protein pairs) specific to *S.* Typhi infection.

Among the hubs, which were reported to have strong relevance to *Salmonella* infections (as indicated by more than one PubMed hits) and found to be required for maintaining the subnetwork stability, hubs CCR1 and IRS2 were considered for further investigation for their condition-specific dynamic features during *S.* Typhi infection (Figure 2 and Figure 3). After constructing a subnetwork with CCR1 as the hub and its interacting partners as nodes, the PCC values for all interactions were integrated and their condition-specific dynamic properties were investigated following labelling the edges with the respective PCC values of individual interactors during different perturbances (Table S5). As indicated in Figure 2, network of the interacting partners of CCR1 represents the dynamicity of the network properties during *Salmonella* Typhi infection. Out of its 18 interactors as per PPI from HPRD, CCR1 and its four interactors, which include CCL15, CCL26, CCL8 and TPST1 showed unique correlated expression patterns (lower PCC values corresponding to poorly correlated expression between protein pairs) specific to *Salmonella* Typhi infection (Table S8). In comparison to the controls (healthy) and other infections, decreasing PCC values of the above interactors of CCR1 hub indicate that they are loosely correlated during *Salmonella* Typhi infection. Larger values of PCC indicate higher correlation between evaluated gene pairs. Thus, the expression of CCR1 is strongly correlated with the expression of its partners in normal/convalescent phase of *Salmonella* Typhi infection. Dynamic nature of the salmonella-specific subnetworks was also apparent by the changes in the PCC of interactions in other disease conditions studied (data not shown). Similarly, dynamic nature of the hubs IRS2 (Figure 3 and Figure S3) and PRKCA (Figure S2) with their interacting partners was also investigated. Five interacting partners, such as IL4R, JAK3, PIK3CD, SHC1 and TYK2 of the hub IRS2 are also loosely bonded when *Salmonella* Typhi infects the human host as indicated by the lower PCC values (Table S9). There exists no previous report where direct involvement of IRS2 protein of the human host during *Salmonella* Typhi infection was mentioned.

## Discussion

In this study, *S.* Typhi infection-specific subnetworks were identified by integrating human gene expression profiles during *S.* Typhi and other bacterial infections and complex diseases like cancer. This comparative analysis was also adopted to determine the dynamic nature of the PPI network unique to *S.* Typhi infection. Other Gram-negative bacteremic infections (*Acinetobacter*, *Klebsiella*, and non-typhoidal *Salmonella*) and *Escherichia coli*-infected expression profiles were included to compare the host expression levels during other pathogenic and phylogenetically-affiliated bacterial infections. Similarly, gene expression profile of human peripheral blood cell during Gram-positive (*Streptococcus pneumonia*) bacteremic infections were considered to evaluate the differences in the expression patterns because of the variation in the outer casing of the bacteria. To make sure that the changes of host gene expression are due to bacterial infection, one expression profile of Acute Myeloid Leukemia (AML) [25] was also included in this study. Leukemia gene expression data was used as negative control dataset to exclude host gene expression changes due to non-infectious diseases. Thus, comparison of this dataset with the expression datasets of different bacteremic infections helped us to identify the PIN unique to bacterial infection. All these expression datasets were originally generated to evaluate the infection- and disease-related changes in gene expression and relevant biological processes related to a particular infection and/or disease. In this study, we only focused on the changes in protein-protein interaction patterns specific to *Salmonella* Typhi infection based on the expression profiles.

Protein-protein interaction networks are static, as they include all possible interactions regardless of when or where the interactions take place. The integration of expression data and the PPI network allowed us to identify functionally-important genes, which were absent in the list of significantly differentially-expressed genes in the expression data analysis carried out previously [26], [18], [37].

A number of microarray expression data for the host and the pathogen (*S.* Typhi) are available for human typhoid fever. However, more focus should be given to find out ways to integrate such information in order to gather disease-specific knowledge and therapeutic targets as well as their reproducibility using different data sources. Few previous investigations addressed this problem by integrating gene microarray datasets to investigate the nature of subnetworks or co-expression in heart failure [19] [20], dilated cardiomyopathy [26], breast cancer [18] and aging [17]. Similarly, we attempted to identify the uniquely-altered co-expression subnetworks during *Salmonella* Typhi infection and characterize their dynamic nature. A potential error that may arise due to the so called “batch effect” is not applicable to our study because of two reasons: i) Each GEO series dataset used in our study was run in one platform only, with small sample size (varies between 6–16) and in a single batch and ii) we computed the differential expression profile of the hub proteins and the neighbouring nodes (Pearson Correlation Coefficient (PCC)) based on the dynamic protein-protein interaction network constructed with single microarray gene expression dataset. Thus, we did not combine independently run microarray datasets from different experiments that give rise to the batch effect. Each microarray dataset considered for our study was from a single experiment. Our investigation is in line with the previously established methods, where analysis primarily relied on the PCC values calculated from the gene expression data to define condition-specific subnetworks from most frequently-used curetted PPI dataset retrieved from the HPRD database [21]. Pearson Correlation Coefficient (PCC) was widely used as the similarity measure between the expression profiles of the genes, which encode the interacting proteins, in spite of its known limitation to measuring the strength of only linear relationships. It is well known that protein pairs encoded by co-expressed genes interact with each other more frequently than the random pairs. The similarity in the mRNA expression profiles has been associated with biologically relevant PPIs [46]. In the present work, we computed the PCC between the expression profiles of the genes whose corresponding proteins are known to interact [47]. To determine whether mRNAs corresponding to interacting protein pairs are likely to be co-expressed, we used PCC of the corresponding gene pairs in all the studied conditions. It is also to mention that PCC measures the relative shape of the expression profile rather than its expression values. In addition to that, other reasons for usefulness of PCC for co-expression studies are reported by Mentzen and Wurtele 2008 [48] and Daub et al 2004 [49]. They estimated that the presence of strong non-linear relationships between gene expression profiles in the expression data, which would not be picked up by Pearson's R, is relatively rare. The PCC is easy to calculate and is familiar to experimental biologists [50]. Gustin et al 2008 [51] indicated that genes are usually said to be co-expressed or connected when their expression levels are linearly linked within a group. The PCC remains the basic method for assessing pairwise expression of linearly-linked genes.

CCR1, a chemokine (C-C motif) receptor, is one of the hubs of *S. Typhi* infection-specific dynamic subnetworks. Significantly-enriched pathways in KEGG represented by this hub and its interactors include cytokine-cytokine receptor interaction and chemokine signalling pathway, while the same in REACTOME are chemokine receptors bind chemokines and regulation of IFNγ signalling. Polymorphism in the CCR1 region is associated with coeliac disease in humans [38] and *Salmonella* infection in pig [39]. This is the first study to show an association between CCR1 and *Salmonella* Typhi infection in humans. There are no reports where the direct involvement of IRS2 protein of human host during *Salmonella* Typhi infection was evidenced. Previous studies revealed that IL-4 (Interleukin 4) receptors (IL4R, one of the interacting partners of IRS2) are involved in auto and/or trans-phosphorylation of Janus kinases 1 and 3 (JAK1 and JAK3) and activation of the IL-4 signalling pathways [40], [41]. These pathways include signal transducer and activator Insulin receptor substrate 2 (IRS2) [42], [43]. It was also reported that IL-4 and IRS2 play a critical role in the regulation of immune responses [44] and the pathogenesis of inflammatory bowel diseases.

Our results suggest that disorganization of CCR1 and IRS2 by the loss of co-ordinated co-expression components is associated with *S.* Typhi infection. Thus, changes in the dynamic network modularity that are associated with *S.* Typhi infection may provide a prognostic signature, which may help to identify the molecular markers of human typhoid fever.

## Conclusion

This network-based comparative analysis approach integrates protein-protein interactions with gene expression profiles to reveal dynamic nature of the network under different biological states. The dynamic features of identified *S.* Typhi infection-specific subnetworks may account for the underlying disease mechanisms. The molecular modules identified might be used as potential drug targets and provide new directions for further clinical validation and understanding of the diseases at the cellular level.

## Supporting Information

Figure S1 **The 81 selected genes are important to dynamic subnetworks stability.** Attacking the genes in this subnetwork decreases the CLP of the network.
(TIF)

Figure S2 **Network of the interacting partners of PRKCA representing differences in dynamic network properties in** ***Salmonella*** **infection.** The edges were labelled with respective PCC values of individual interactors during different perturbances. The conditions mentioned are A. Control (normal host cell), B. *Salmonella* infection, C. other bacteremic (non-typhoid *Salmonella*, *Klebsiella* spp and *Acinetobacter* spp) infection, D. Ecoli infection, E. *Streptococcus pneumoniae* and F. Leukemia. As indicated PRKCA and few interactors (e.g, ADRBK1, ANXA7, ARHGEF1, CBL, CISH, CORO1B, DLG4, EWSR1, GFAP, GFPT1, HES1, HLA-A, MBP, MYOD1, NOS1, PLD2, PRKCZ, PTGIR, RHO, RRAD, TERT, TIAM1 and TRIM29) showed unique expression patterns (lower PCC values corresponding to correlated expression between protein pairs) specific to *S.* Typhi infection.
(TIF)

Figure S3 **Network of the interacting partners of IRS2 representing differences in dynamic network properties in** ***Salmonella*** **infection.** The edges were labelled with respective PCC values of individual interactors during different perturbances. The four mentioned conditions are A. Control (normal host cell), B. *Salmonella* infection, C. other bacteremic (non-typhoid *Salmonella*, *Klebsiella* spp and *Acinetobacter* spp) infection, D. E.coli infection, E. *Streptococcus pneumoniae* and F. Leukaemia. Hub IRS2 and four interactors (e.g, IL4R, JAK3, PIK3CD, SHC1 and TYK2) showed unique expression patterns (lower PCC values corresponding to correlated expression between protein pairs) specific to *S.* Typhi infection.
(TIF)

Table S1 **List of common hubs present in all the studied datasets with their respective Avg. PCC.**
(XLSX)

Table S2 **The list of common interactors present in all the studied datasets with their respective PCC.**
(XLSX)

Table S3 **Significantly enriched gene ontology biological process term of 81 hubs and their interactors using DAVID database.**
(DOCX)

Table S4 **Biological pathway analysis of 81 hubs and their interactors by ClueGO (KEGG_24.05.2012 and REACTOME_10.07.2012).** Only those enriched Biological pathways GOTerm were selected that showed low Pvalue (Pvalue ≤0.05).
(DOCX)

Table S5 **List of enriched pathway term (analysed using REACTOME databases) that showed higher number of hits when searched in PubMed with the subject “pathways term and** ***Salmonella*** **/** ***Salmonella*** **Typhi.”**
(DOCX)

Table S6 **List of enriched pathway term (analysed using KEGG databases) that showed higher number of hits when searched in PubMed with the subject “pathways term and** ***Salmonella*** **/** ***Salmonella*** **Typhi.”**
(DOCX)

Table S7 **Pearson Correlation Coefficient (PCC) values of hubs IRS2 with its interactors.**
(XLSX)

Table S8 **Degree of** ***Salmonella*** **Typhi infection specific.**
(XLSX)

Table S9 **Pearson Correlation Coefficient (PCC) values of hubs CCR1 with its interactors.**
(XLSX)
