# Supplementary figures and images for: Dynamic Modularity of Host Protein Interaction Networks in Salmonella Typhi Infection
Source: PLoS One. 2014 Aug 21;9(8):e104911. doi: 10.1371/journal.pone.0104911 (PMC4140748; doi:10.1371/journal.pone.0104911)

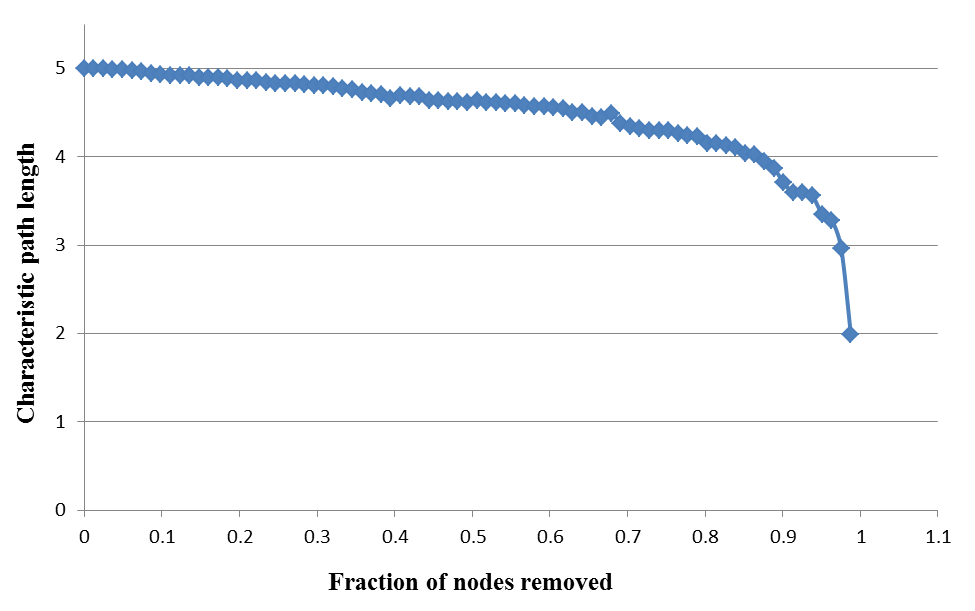

Supplement: Figure S1 — The 81 selected genes are important to dynamic subnetworks stability. Attacking the genes in this subnetwork decreases the CLP of the network. (TIF) [file pone.0104911.s001.tif]

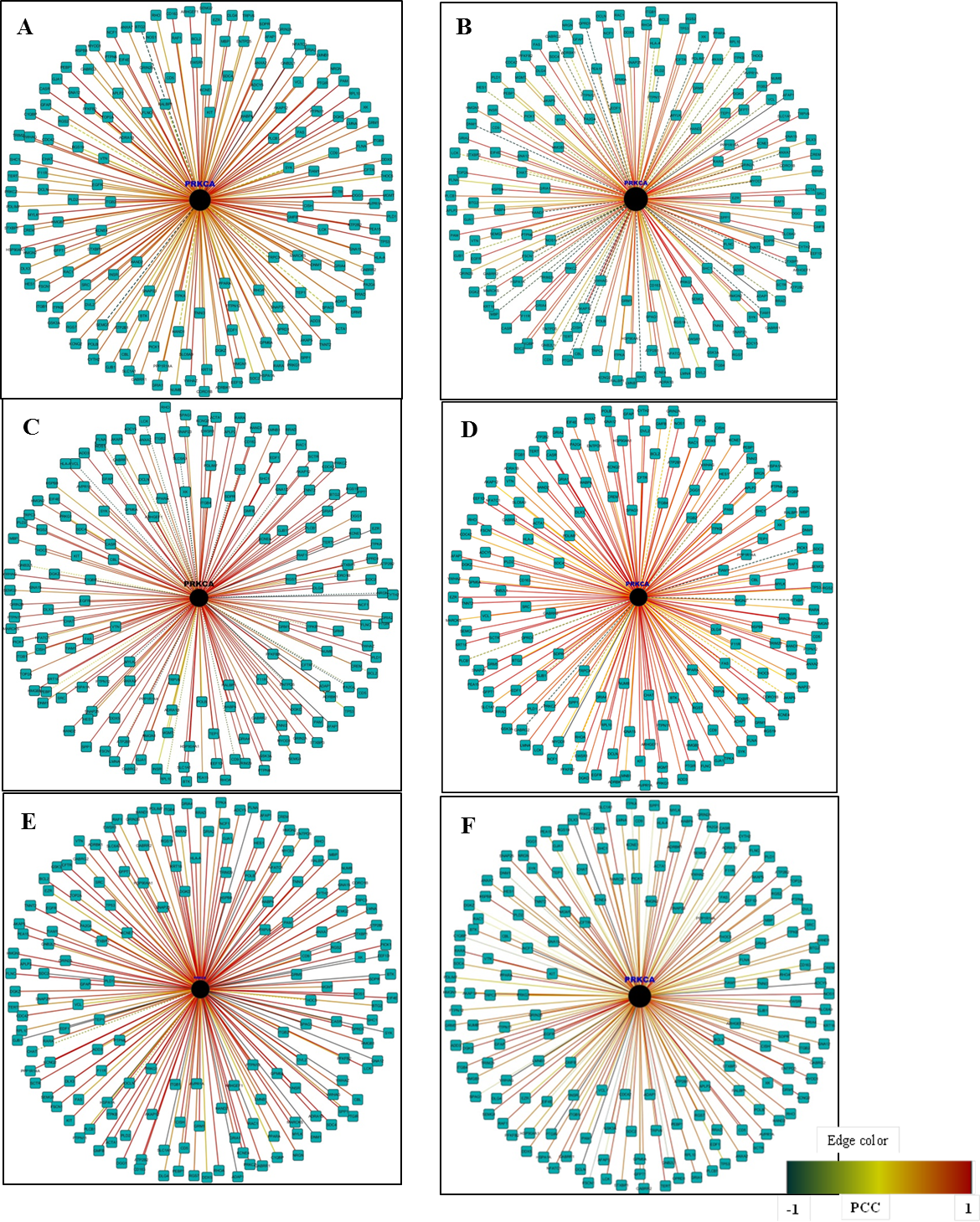

Supplement: Figure S2 — Network of the interacting partners of PRKCA representing differences in dynamic network properties in Salmonella infection. The edges were labelled with respective PCC values of individual interactors during different perturbances. The conditions mentioned are A. Control (normal host cell), B. Salmonella infection, C. other bacteremic (non-typhoid Salmonella, Klebsiella spp and Acinetobacter spp) infection, D. Ecoli infection, E. Streptococcus pneumoniae and F. Leukemia. As indicated PRKCA and few interactors (e.g, ADRBK1, ANXA7, ARHGEF1, CBL, CISH, CORO1B, DLG4, EWSR1, GFAP, GFPT1, HES1, HLA-A, MBP, MYOD1, NOS1, PLD2, PRKCZ, PTGIR, RHO, RRAD, TERT, TIAM1 and TRIM29) showed unique expression patterns (lower PCC values corresponding to correlated expression between protein pairs) specific to S. Typhi infection. (TIF) [file pone.0104911.s002.tif]

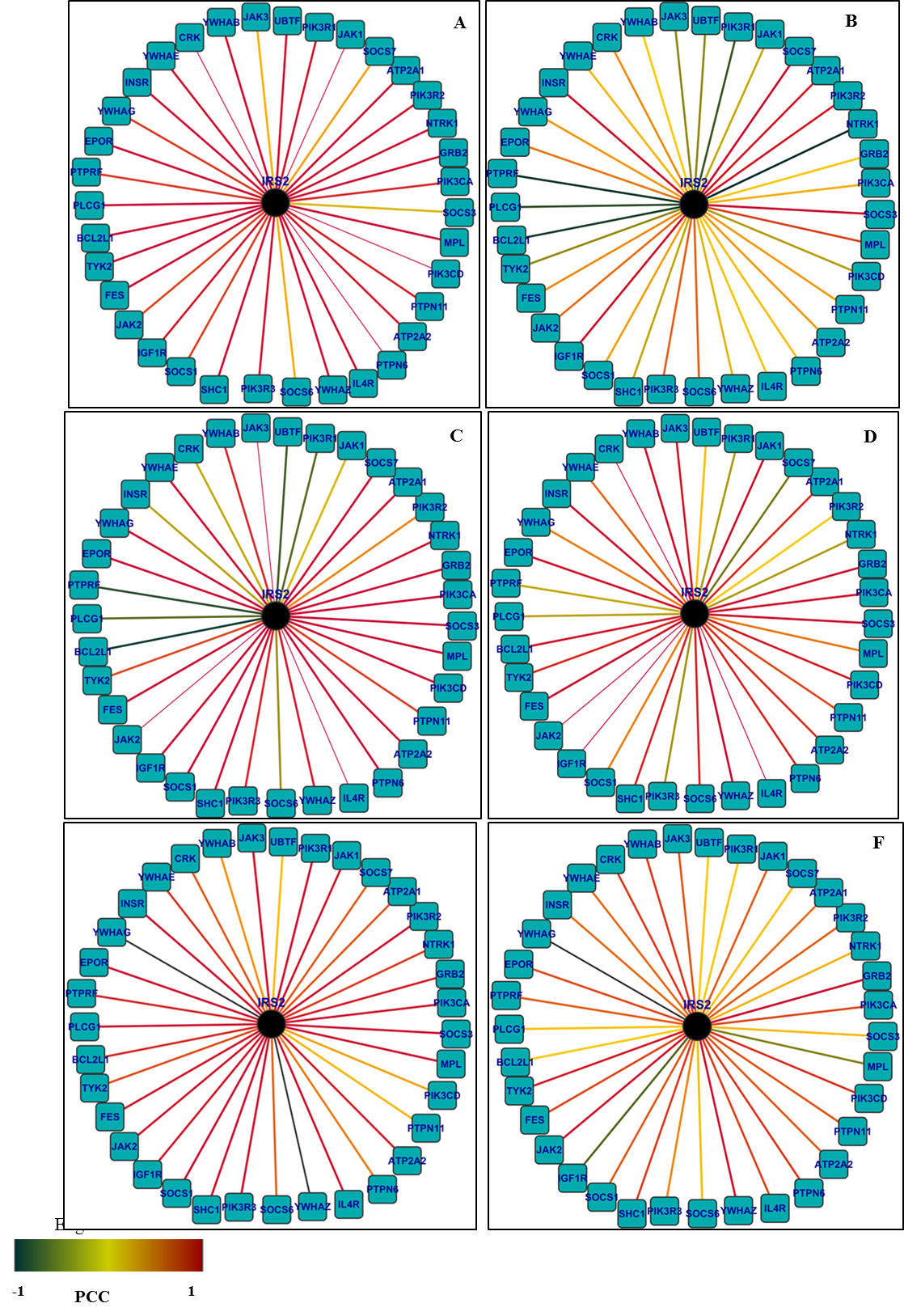

Supplement: Figure S3 — Network of the interacting partners of IRS2 representing differences in dynamic network properties in Salmonella infection. The edges were labelled with respective PCC values of individual interactors during different perturbances. The four mentioned conditions are A. Control (normal host cell), B. Salmonella infection, C. other bacteremic (non-typhoid Salmonella, Klebsiella spp and Acinetobacter spp) infection, D. E.coli infection, E. Streptococcus pneumoniae and F. Leukaemia. Hub IRS2 and four interactors (e.g, IL4R, JAK3, PIK3CD, SHC1 and TYK2) showed unique expression patterns (lower PCC values corresponding to correlated expression between protein pairs) specific to S. Typhi infection. (TIF) [file pone.0104911.s003.tif]
