# Supplementary material for: Dynamic Modularity of Host Protein Interaction Networks in Salmonella Typhi Infection
Source: PLoS One. 2014 Aug 21;9(8):e104911. doi: 10.1371/journal.pone.0104911 (PMC4140748; doi:10.1371/journal.pone.0104911)
Supplement: Table S3 — Significantly enriched gene ontology biological process term of 81 hubs and their interactors using DAVID database. (DOCX) [file pone.0104911.s006.docx]

Table S3: Significantly enriched gene ontology biological process term of 81 hubs and their interactors using DAVID database.

| **Hub** | **Interactors** | **GO Term** | **FDR** |
| --- | --- | --- | --- |
| ARCN1 | COPZ2, COPB2, COPA, COPG2, COPB1, ARCN1, COPG | GO:0006886~intracellular protein transport | 3.69 × 10^-07^ |
| ARHGDIG | CDC42, ARHGDIG, RAC1, RHOA, RHOB, RHOG, RHOH | GO:0007264~small GTPase mediated signal transduction | 1.6 × 10^-07^ |
| ARPC5 | ARPC5, ACTR3, MAPKAPK2, ARPC4, ACTR2, ARPC1B | GO:0006928~cell motion | 0.042 |
| ATG12 | ATG12, PLSCR1, KRTAP4-12, ATG3, ATG7, ATG5, ATG10, PTK2, MDFI | GO:0006914~autophagy | 2.3 × 10^-06^ |
| ATG7 | GABARAPL2, ATG10, ATG12, MAP1LC3B, ATG7, ATG3, GABARAP | GO:0006914~autophagy  GO:0019941~modification-dependent protein catabolic process | 8.0× 10^-10^  0.041 |
| ATP2A2 | ATP2A2, IRS1, IRS2, BCL2, PLN, S100A1, CAMK2A | GO:0048878~chemical homeostasis | 0.044 |
| BAD | BAD, PIM2, BCL2L1, BCL2A1, YWHAH, YWHAZ, YWHAB, BCL2L2, S100A10, RPS6KA3, YWHAG, MAP2K5, YWHAQ, BCL2, SNCA, PRKACA, RPS6KA1, RPS6KA2, HRK, WASF1, YWHAE, BCL2L10, PAK7, MCL1, PPP1CA, PPP3CA, RPS6KA5, EWSR1, SFN, RAF1, MAPK8, PAK1, PIM1, PIM3, AKT1 | GO:0042981~regulation of apoptosis  GO:0043069~negative regulation of programmed cell death | 4.97 × 10^-11^  5.42 × 10^-11^ |
| BMP7 | BMP7, ACVR1, ACVR2A, ENG, BMPR2, BMPR1A, NCOA3, ACVR2B, NOG, BMPR1B, GDF7, CHRDL2, SOSTDC1 | GO:0001934~positive regulation of protein amino acid phosphorylation  GO:0042327~positive regulation of phosphorylation | 1.20E-05  1.85E-05 |
| CAPN3 | LNC, CAPN3, DYSF, NECAB2, YWHAQ, TTN | NA | NA |
| CCL18 | CCL18, UNC119, C14orf1, EEF1A1, TP53, TLE1, CRMP1 | NA | NA |
| CCL2 | DARC, CCL2, VCAN, MMP1, MMP8, MMP3, CCR10, CCR1, CCR2, CCR5, CCRL1, CCBP2 | GO:0006935~chemotaxis | 1.33E-06 |
| CCL8 | CCL8, CCR1, CCR2, CCR3, CCR5, VCAN, MMP3, CCRL1, CCBP2, DARC | GO:0006935~chemotaxis | 2.27E-07 |
| CCR1 | CCL3, CCL4, CCL5, CCR1, PLP2, CREB3, JAK1, STAT1, STAT3, CCL26, CCL2, CCL7, CCL14, CCL15, CCL16, CCL3L1, CCL8, CCL23, GNA14, TPST1, TPST2 | GO:0006935~chemotaxis | 1.8210^-18^ |
| CD27 | CD27,TRAF2,TRAF3,TRAF5,SIVA1,CD70 | GO:0042981~regulation of apoptosis  GO:0022610~biological adhesion | 8.7810^-04^  0.002 |
| CD36 | YES1, LYN, ITGB3, COL1A1, COL1A2, ITGB1, FYN, CD9, ITGA6, THBS1, SRC, MATK, ITGA2B | GO:0022610~biological adhesion | 0.00 |
| CD3E | CD3E,CD3EAP,PIK3R1,ZAP70,CD3D,TRB@,SYK,SHC1,NCK2,TOP2B,CD79B,NCL,UNC119,NCK1,TRAT1,CD3G,PTPN22,LCK | GO:0007166~cell surface receptor linked signal transduction | 1.9010^-04^ |
| CD5 | CD5,CD72,RASA1,CSNK2A1,CD79A,CD79B,FYN,LCK,CBL,PIK3R1,PTPN6,ZAP70,PRKCA,PRKCB,PRKCG,CD6,CD247,CD4,CD2,DYNLT3,HNRNPU,CAMK2D | GO:0007166~cell surface receptor linked signal transduction  GO:0006796~phosphate metabolic process | 0.02  4.8310^-04^ |
| CIT | RHOC,RHOA,GRIN2D,DLG4,DISC1,GRIN1,CIT | NA | NA |
| CNOT8 | CNOT8,BTG2,CNOT3,CNOT4,CNOT1,CNOT6,BTG1,CNOT2 | GO:0045449~regulation of transcription | 0.0101269 |
| COPE | COPA, COPG2, ARF1, COPB1, COPG, COPE | GO:0006890~retrograde vesicle-mediated transport, Golgi to ER | 4.79E-12 |
| CUL4A | CUL4A,CAND1,DDB1,SKP2,CDKN1B,RBX1,CHEK1,DDB2 | GO:0019941~modification-dependent protein catabolic process | 0.003226309 |
| CYBA | CYBA,NCF4,NCF2,RAC1,NOX1,NCF1,CYBB | GO:0042554~superoxide anion generation | 9.76E-09 |
| DNAJA3 | DNAJA3,SMAD9,RAD51,HSPA8,ZBTB22,RASA1,HSPA1A,IFNGR2,JAK2,MET,RNF34,ATXN1,FAM131C,NTRK1,USP7,COIL,GFI1B | NA | NA |
| DTNA | DTNA,GFI1B,DRP2,DMD,SNTB1,SNTB2,SNTA1,UTRN,ACTA1,DTNBP1,SYNM,KCNJ12,SNTG1,SNTG2,SYNC | GO:0006936~muscle contraction | 8.80E-05 |
| DUSP1 | DUSP1,MAPK14,HSPA4,SKP2,CKS1B,MAPK1,UBB,MAPK8,MAPK3,MAPK12 | GO:0007265~Ras protein signal transduction | 0.049 |
| FCGR2B | FCGR2B,PTPN6,INPPL1,LY6E,CRP,C14orf1,INPP5D,APCS,LYN,BLK,MAPK1,MAPK3 | NA | NA |
| FGFBP1 | FGFBP1,FGF1,FGF2,UBQLN4,HSPG2,FIBP | GO:0008543~fibroblast growth factor receptor signaling pathway | 0.033542174 |
| FOS | BCL3, CSNK2A1, CSNK2A2, JUNB, STAT1, SMAD3, ATF2, LMNA, DDIT3, CEBPG, HNF1A, RUNX1, MITF, NFKB1, RELA, ETS1, FOS, SUMO1, SUMO2, SUMO3, SUMO4, ATF7, EEF1D, RB1, SMARCD1, MAP3K7, NCOA1, NFATC3, TSC22D3, RPS6KA4, GATA4, BATF, COBRA1, USF2, NCOR2, EPHB2, PRKACA, PSMC5, RPS6KA1, GTF2F2, XBP1, ELK1, TAF1, TBP, RUNX2, ELK4, USF2, NCOR2, EPHB2, PRKACA, PSMC5, RPS6KA1, SMARCD1, MAP3K7, NCOA1, NFATC3, TSC22D3, RPS6KA4, GATA4, BATF, COBRA1 | GO:0043067~regulation of programmed cell death, GO:0045449~regulation of transcription | 4.34× 10^-17^ |
| GCM1 | GCM1,HDAC5,HDAC1,HDAC4,HDAC3,CREBBP | GO:0016570~histone modification | 3.95E-05 |
| GLS2 | GLS2,SNTA1,PAG1,DLG1,DLG2,DLG3,INADL,RGS3,CASK,TAX1BP3,DLG4 | NA | NA |
| HIST2H2BE | HIST2H2BE,RCC1,PTMA,TGM2,TBL1X,HIRA,KPNA1,HSPD1,GADD45A,LALBA,TNPO1,HIRIP3,SAP30,DYRK2,NAP1L4,BRD7,TBL1XR1,AKT1,ATF2 | NA | NA |
| HOXC8 | HOXC8,JUN,SMAD4,BMPR1A,SMAD1,BTG2,SMAD6,GMNN,HOMEZ | GO:0007179~transforming growth factor beta receptor signaling pathway | 3.33E-05 |
| IL2RA | STAT3,IL2RB,IL2,IL2RA,ICAM1,NFKB1,CD4,STAT5B | GO:0031328~positive regulation of cellular biosynthetic process | 0.009 |
| INADL | KCNJ15, KCNJ10, GRIN2D, SCN4A, CACNG2, PARD3, GRIN2B, GRIN2A, GRIN2C, CLDN1, NLGN2, MPP5, ACCN3, NRXN2, CRB3, TJP3, CRIPT, KCNA4, CNKSR2, KIF1B, MAPK12, SCN5A, GLS2, KCNJ2, PAX6, PRDM16, DDX18 | GO:0015672~monovalent inorganic cation transport | 5.24× 10^06^ |
| INHBB | INHBB,ACVR1,ACVR2A,INHBA,INHA,INHBB,IGSF1,INHBC,ACVR1B,ACVR2B,ACVR1C | GO:0032925~regulation of activin receptor signaling pathway | 1.42E-08 |
| IRS2 | IRS2,PIK3R1,PLCG1,PTPN11,TYK2,ATP2A1,BCL2L1,JAK3,SHC1,UBTF,PTPN6,PIK3CD,PIK3R2,SOCS1,PIK3R3,SOCS7,NTRK1,EPOR,IGF1R,IL4R,JAK1,PTPRF,GRB2,YWHAZ,YWHAE,SOCS6,YWHAG,SOCS3,JAK2,ATP2A2,MPL,CRK,PIK3CA,FES,YWHAB,INSR | GO:0006793~phosphorus metabolic process | 7.66E-05 |
| JUN | JUN, BCL3, BCL6, BRCA1, STAT3, TOP1, MAPK11, ELF3, NCOA2, ATF1, ATF2, MAPK10, DDIT3, TOP2A, EGR1, TCF4, SNAPC5, ESR1, FOSL1, NR3C1, NFE2L2, HOXA9, HOXC8, RUNX1, MYOD1, HNRNPM, NFE2L1, RELA, ETS1, ETS2, SKI, FOS, SPI1, POU1F1, MAF, RB1, NR5A1, NFYA, SP1, GTF2F2, ABL1, TPM1, HCFC1, TAF1, AR, TBP, CREBBP, RUNX2, STAT1, STAT4, HMGA1, PRKDC, SMAD4 PIN1, MAPK8, NACA, SMAD2,TDG, FOSL2, SMARCD1, VDR, MAPK3, SUMO1, NCOA3, NRIP1, TGIF1, NCOA1, EP300, MAPK9, TSC22D3, TCF20, SMAD3, DHX9, ATF3, HIF1A, TRIP4, MYBBP1A, PRKD1, DDX21, SPIB, PACS1, ATF4, HHEX, COPS5, MAFB, RFWD2, EN1, GTF2B, HDAC3, UBE2I, RBM39, RAGE, MAPKAPK5, BATF, CREB5, COBRA1, TPM2, PIAS1, RPL18A, JDP2, SNRK, SUMO2, SUMO3, FBXW7, CSNK2A1, ERG, SOX8,SOX10, SUMO4, ITCH, SMARCD3, NTRK3, IRAK1, ELOF1, SNAPC5, GATA2,UBB | GO:0045449~regulation of transcription, GO:0016310~phosphorylation, GO:0042981~regulation of apoptosis | 1.04× 10^35^ |
| JUNB | JUNB,BCL6,BRCA1,JUNB,NINL,FOSL1,NFE2L1,FOSB,MAPK14,SMAD4,MAPK8,FOSL2,SMAD3,BATF,JDP2,FOS,ESR1 | GO:0006355~regulation of transcription, DNA-dependent | 5.93E-08 |
| KCNK15 | KCNK15,YWHAG,YWHAH,YWHAE,YWHAB,YWHAZ,YWHAQ,SFN | GO:0006886~intracellular protein transport | 4.00E-04 |
| KCNK3 | KCNK3,COPB1,YWHAB,S100A10,YWHAZ,YWHAQ,YWHAE,YWHAH,YWHAG,SFN | GO:0006886~intracellular protein transport | 3.68E-06 |
| KRT19 | KRT19,TUBG1,KRT15,KRT6B,PCM1,FANCG,HGS,ABI2,FAM107A,EXOC8,SFI1,ZNF638,C10orf10,DGCR6L,USHBP1,DMD,PNN | NA | NA |
| LIMK2 | LIMK2,LIMK1,CFL1,GSN,ROCK1,CDC42BPA,PARD3,HIPK3 | GO:0030036~actin cytoskeleton organization | 0.003 |
| MTA2 | MTA2,SATB1,RBBP7,RBBP4,MBD3,TP53,SPEN,APPL1,APPL2 | GO:0045892~negative regulation of transcription, DNA-dependent | 8.34E-04 |
| MKNK1 | MKNK1, MAPK1, MAPK14, EIF4G3, PLA2G4A, EIF4G2, EIF4E, MBP, MAPK3. EIF4G1  PAK2 | GO:0006417~regulation of translation  GO:0010608~posttranscriptional regulation of gene expression  GO:0032268~regulation of cellular protein metabolic process | 3.20E-05  2.78E-04  0.015 |
| MUC7 | SELL,MUC7,LTF,GALNT10,GALNT14,GALNT12,HTN3,AMY1A | NA | NA |
| NCF4 | TXN,NCF2,MSN,CORO1A,XRCC6,PRKCD,CYBA,NCF1,CYBB | GO:0042554~superoxide anion generation  GO:0006800~oxygen and reactive oxygen species metabolic process | 7.48E-05  0.012 |
| NEK9 | RAN,NEK6,NEK7,BICD2,NEK9,SSRP1,SUPT16H,NEK9,MBP | NA | NA |
| NOTCH3 | NOTCH3,PSEN2,MAML2,MAML3,CHUK,RBPJ,JAG1,JAG2,SNW1,MAML1,DLL1,KAT2B,PSEN1 | GO:0007219~Notch signaling pathway  GO:0007166~cell surface receptor linked signal transduction | 2.72E-17  0.003 |
| PGF | NRP2, FLT1, NRP1, PGF, VEGFA | GO:0001525~angiogenesis | 1.80E-05 |
| PLEKHB1 | SMAD1,ACVR1,BMPR1B,GNGT1,PLEKHB1,TGFBR1 | GO:0007178~transmembrane receptor protein serine/threonine kinase signaling pathway | 0.006 |
| PNKP | PNKP,XRCC1,KRTAP4-12,XRCC4,SMAD5,MAGEA11,TRIM37,MYOZ1 | NA | NA |
| PPP2R3A | PPP5C,ATXN7L2,RBL1,PPP2R3A,HMGB1,AKAP9,CDC6 | NA | NA |
| PRKCA | KIT, OPRD1, RHOA, SPP1, PAM, PFKFB2, KCNE1, PTPN11, PRKCA, RRAD, RHO, DDX5, SRC, TP53, VCL, VTN, NCF1, GJB1, RPL10, ITGB2, PTPN12, SNAP25, NFATC1, DLX3, CASR, GPM6A, DGKZ, ADD3, GMFB, PA2G4, NRGN, DNM1, PLD1, HAND1, CFTR, CYTH2, RGS7, SNAP23, OCLN, STXBP1, SPAG1, PEA15, GRM1, RGS19, CD163, TRPV6, GSK3A, PLCB1, HSPB8, PEBP1, CHAT, MARCKS, PRKG1, PTGIR, EDF1, ADAP1, TNNI3, HABP4, GABRR1, TRPC3, PPP1R14A, THOC5, GRIA2, HMGB1, RARA, SDC4, TNNT2, ATP2B1, ADRBK1, PTPN6, SLC6A9, KCNQ2, GRM5, F11R, CDC42, ATP2B2, CBL, SHC1, DGKD, ACTA1, GJA1, DVL2, CREM, CORO1B, EZR, TOP2A, FLNC, RALBP1, EEF1D, EGFR, EIF4E, EWSR1, SLC1A1, ITGB1, GABRG2, GFAP, GRIA4, GRIA1, GRIN2B, GRIN2A, GFPT1, GNA15, HES1, HSPA1A, SDC2, HLA-A, CD9, ADRA1B, ITPKA, ITPKB, INSR, KRT18, LMNA, LMNB1, BCL2, ANXA2, CD5, LCK, MGMT, MBP, MYOD1, NOS1, APLP2, HMGN2, HMGN1, RAF1, GNB2L1, PRKCZ, SCTR, SEMG1, SEMG2, TERT, XK, SYK, TIAM1, AVPR1A, RGS2, MYLK, C1QBP, YWHAZ, BTG2, TEP1, ARHGEF1, OGG1, RAC1, PLD2, HAND2, CISH, FSCN1, DLG4, GNA12, AKAP5, AKAP12, YWHAG, POLB, ADCY5, SDPR, KCNE4, AFAP1, TRIM29, PDLIM7, STXBP3, GABRR2, BTK, ANXA7, FAS, ITGB4, PICK1, PPARA, FLNA, NUMB, HSP90AA1, ENTPD5 | GO:0044093~positive regulation of molecular function, GO:0043549~regulation of kinase activity, GO:0043067~regulation of programmed cell death | 6.77× 10^06^ |
| PRMT2 | RXRA,NCOA6,PGR,THRB,DMRTB1,CPSF7,PRMT2,RB1,ESR1,BAT2,HNRNPUL1,ESR2,NCOA1 | GO:0045449~regulation of transcription | 0.018 |
| PVRL3 | PVR, PARD3, PVRL1, PVRL3, PVRL2, MLLT4 | GO:0007155~cell adhesion | 4.35E-04 |
| PYCARD | PYCARD,MEFV,SRF,NLRP3,NLRC4,CASP1,NLRP12,PRKAR1A,NLRP1,PYDC2,POP1 | GO:0006919~activation of caspase activity | 3.71E-05 |
| RALB | RALB,CALM1,REPS2,RALBP1,REPS1,RGL4,PLCD1 | NA | NA |
| RALBP1 | RALBP1,RALA,RALB,REPS2,AP2M1,RAC1,HOOK2,REPS1,CCNB1,SYNJ2BP,IKBKG,CASP8,TRAF2,FADD,PRKCA | GO:0043065~positive regulation of apoptosis  GO:0043068~positive regulation of programmed cell death | 0.03  0.03 |
| RANBP2 | RANBP2,OPN1MW,OPN1LW,RAN,HDAC4,MDM2,IPO5,RANGAP1,XPO1,KPNB1,TNPO1,XPOT,NUP62,UBE2I,PARK2 | GO:0006913~nucleocytoplasmic transport | 4.64E-06 |
| RARG | RARG,SMAD3,ITGB1BP2,MAP6,NR0B2,PNRC1,PNRC2,RXRB,RXRA,RXRG,NCOR2,NCOA3,NCOA1,HMGA1 | GO:0045449~regulation of transcription | 1.78E-04 |
| RASGRP2 | RASGRP2,KRAS,FAM118B,PTCHD2,NRAS,RAP1A | GO:0007264~small GTPase mediated signal transduction | 0.02 |
| RHOB | RHOB,PGGT1B,PDE6D,ARHGDIG,RHPN2,ARHGEF3,PPP2CA,FNTA | NA | NA |
| RND2 | RND2,RACGAP1,UBXN11,FNBP1,VPS4A,MEOX2 | NA | NA |
| RRN3 | RRN3,TAF1B,TAF1C,POLR1B,POLR1A,EIF3L,MYO1C,TAF1B,TAF1C,POLR1B,POLR1A,EIF3L,MYO1C | NA | NA |
| S100P | AGER,S100P,EZR,S100A4,S100A2,CACYBP,S100Z,SUGT1,S100A1 | NA | NA |
| SCAP | SREBF2,SCAP,INSIG1,INSIG2,PGRMC1 | GO:0030967~ER-nuclear sterol response pathway | 9.96E-05 |
| SERPINB6 | SERPINB6,PLG,PROC,F2,PLAU,F10,KLK2 | GO:0006508~proteolysis | 0.003 |
| SERPINC1 | SERPINC1,SDC2,KLK2,PLG,F2,KLK6 | GO:0042060~wound healing | 0.013 |
| SFN | SFN,ZFP36,TSC2,BAX,TRIM25,CDK1,MARK3,BAD,CHST1,WDYHV1,TBL3,PLK4,GPRIN2,PLEKHF2,FAM189A2,KIAA0408,MAP3K5,HNRNPD,EIF2S1,EIF4B,EEF1A1,NR3C1,BCR,HDAC5,CCAR1,MST1R,YWHAG,CDC25B,ING1,KCNK3,KCNK9,KCNK15,EGFR,ABL1,MDM4,RFFL | NA | NA |
| SKP2 | SKP2,UBB,WEE1,E2F1,CDKN1B,CDKN1C,MYBL2,SKP1,CKS1B,CDC34,CDK2,CCNA2,CCNE1,RB1,TCF3,ORC1L,CCNT1,CUL1,PFDN1,CDT1,GPS1,CDK9,FZR1,CUL4A,DDB1,MYC,MYB,NLK,DUSP1,TAL1,ELF4 | GO:0007049~cell cycle | 1.96E-18 |
| SLC9A3R2 | SLC9A3R2,PTH1R,RDX,SLC9A3,ATP2B2,TAZ,PLCB3,ADRB2,P2RY1,PRKACA,SIAH1,CFTR,PODXL,SGK1,ACTN4,PDPK1,EZR,SLC22A4,SLC22A5,SLC22A9,SLCO1A2,LPAR1,SGK3,SRY,SLC34A1,KCNJ1,WWTR1,ADORA2B,TBC1D10A,SLCO3A1,PTEN,LPAR2 | NA | NA |
| SMG5 | SMG5,TERT,UPF1,SMG1,SMG6,SMG7 | GO:0000184~nuclear-transcribed mRNA catabolic process, nonsense-mediated decay | 1.01E-07 |
| SNAPC5 | SNAPC5, SNAPC4, JUN, NMI, NOL4, BRCC3, BATF, JUN | NA | NA |
| SOCS7 | SOCS7,PLCG1,GRB2,NCK1,SORBS3,EGFR,PIK3R1,IRS2,TUBA1A,TUBB2C,PIK3R2,IRS4 | GO:0007169~transmembrane receptor protein tyrosine kinase signaling pathway | 3.87E-04 |
| TCEB3B | TCEB3B,TCEB2,TCEB1,ZNF165,TRAF2,DVL2,CBX5,TRIM37,CALCOCO2,SOHLH2,ZBTB43,KRTAP4-12,C19orf50,CEP70,COIL | NA | NA |
| TFF1 | TFF1,GKN2,UBQLN1,UBQLN4,MUC6 | NA | NA |
| TNPO1 | SRP19,NUP98,RAN,RANBP2,HNRNPD,HIST2H2BE,PABPN1,NXF1,RGPD5,HIST3H3,TNPO1,NUP214,HNRNPA1,RPL5,RPS7,NUP153,RPL23A,ELAVL1 | GO:0050658~RNA transport | 1.41E-10 |
| UBE2B | UBE2B,CRMP1,UNC119,UBR1,MED31,RAD18,UBR2,CNOT4 | GO:0019941~modification-dependent protein catabolic process | 0.049 |
| UBQLN4 | UBQLN4,SERPINH1,ATXN1,DTX2,C1QTNF1,ERP27,ZG16B,SMCR7,ITPRIPL1,CCDC107,ANKRD13D,NOTCH2NL,NOMO3,UBQLN4,PMEPA1,PELI2,PBXIP1,MIF4GD,KLHDC5,SCAF1,RIC8A,MOAP1,CCDC136,CCDC14,RSRC2,C11orf49,FA2H,CCDC134,CCDC33,GKAP1,ADAM33,ADPGK,USMG5,C1orf94,ATPIF1,SMAD9,EDN1,ELF5,FKBP2,GABRD,GDI1,GPX7,HK2,IGFBP6,IGHM,IGLC1,IMPDH2,STMN1,COL8A1,COPB1,MDK,CD99,MLLT6,NME3,NPHP1,NPPA,OAT,SERPINE1,PCDH8,SERPINI2,PIN1,PPIB,PPIC,SRGN,QSOX1,PTPRN,PTPRN2,RAD23A,ROBO2,RPN1,CSTF2,DMPK,EEF1A1,PTN,RXRA,UNC119,CCL21,SEMG1,SFRS2,SCG5,SPINT1,SPP1,HSPA13,TFF1,TGFB1I1,TRAF2,UROS,VIP,LAT2,ZNF205,SCG2,AREG,BAT3,SMARCB1,NAE1,ZBTB22,MDM2,RBM10,FZD7,PIP4K2B,CACNA1G,HGS,PNMA1,PDLIM7,CRIPT,PICK1,DAZAP2,FGFBP1,ARL4C,STAM2,NXF1,AGR2,RAI2,RNPS1,PDIA5,ERP29,YWHAQ,IMMT,CPSF6,PLA2G16,TRIM32,SCMH1,TNRC6B,CSTF2T,ZFPM2,NOMO1,SPAG8,HAVCR1,DKK3,PCDH17,TRIB2,UBQLN2,UBQLN1,EFEMP2,CEND1,PLUNC,ZDHHC3,WAC,CYB5R1,EPDR1,UBR7,KLHL26,FAM48A,PRPF40A,EAPP,C19orf10,RNF11,RUNX1T1,CDSN | NA | NA |
| USP4 | USP4,RB1,TRIM21,NOLC1,RBL1,TP53BP2 | NA | NA |
| ZFP36 | ZFP36,YWHAH,NUP214,CCDC85B,HMGB1,MAPK1,YWHAB,SFN,YWHAG,EXOSC8,UPF2,MAPKAPK2,EXOSC6,XRN1,EDC3 | NA | NA |
