# Supplementary material for: Dynamic Modularity of Host Protein Interaction Networks in Salmonella Typhi Infection
Source: PLoS One. 2014 Aug 21;9(8):e104911. doi: 10.1371/journal.pone.0104911 (PMC4140748; doi:10.1371/journal.pone.0104911)
Supplement: Table S4 — Biological pathway analysis of 81 hubs and their interactors by ClueGO (KEGG_24.05.2012 and REACTOME_10.07.2012). Only those enriched Biological pathways GOTerm were selected that showed low Pvalue (Pvalue ≤0.05). (DOCX) [file pone.0104911.s007.docx]

Table S4: Biological pathway analysis of 81 hubs and their interactors by ClueGO (KEGG_24.05.2012 and REACTOME_10.07.2012). Only those enriched Biological pathways GOTerm were selected that showed low Pvalue (Pvalue ≤ 0.05)

| **Hub** | **Interactors** | **REACTOM** | | **KEGG** | |
| --- | --- | --- | --- | --- | --- |
|  |  | **Pathways** | **Term P_Value_** | **Pathways** | **Term P_Value_** |
| ARCN1 | COPZ2, COPB2, COPA, COPG2, COPB1, ARCN1, COPG | 1.COPI Mediated Transport  2.Golgi to ER Retrograde Transport | 2.91E-15  2.91E-15 |  |  |
| **ARHGDIG** | CDC42, ARHGDIG, RAC1, RHOA, RHOB, RHOG, RHOH | Signaling by Rho  GTPases Rho GTPase cycle | 9.57E-13  9.57E-13 | **1.Bacterial invasion of epithelial cells**  2. Shigellosis  **3.Adherens junction** | 7.26E-08  8.82E-06  1.52E-05 |
| ARPC5 | ARPC5, ACTR3, MAPKAPK2, ARPC4, ACTR2, ARPC1B |  |  | **1.Bacterial invasion of epithelial cells**  2.Pathogenic Escherichia coli infection  3.Shigellosis | 5.40E-06  2.59E-06  3.55E-06 |
| ATG12 | ATG12, PLSCR1, KRTAP4-12, ATG3, ATG7, ATG5, ATG10, PTK2, MDFI | Negative regulators of RIG-I/MDA5 signaling | 1.56E-04 | Regulation of autophagy | 3.69E-09 |
| ATG7 | GABARAPL2, ATG10, ATG12, MAP1LC3B, ATG7, ATG3, GABARAP |  |  | Regulation of autophagy | 3.57E-12 |
| ATP2A2 | ATP2A2, IRS1, IRS2, BCL2, PLN, S100A1, CAMK2A | 1.Signal attenuation  2.SOS-mediated signalling  3.IRS activation  4.PI3K/AKT activation  5.Growth hormone receptor signaling | 5.91E-05  4.48E-05  1.38E-05  3.81E-04  1.36E-04 | Type II diabetes mellitus  Aldosterone-regulated sodium reabsorption | 8.56E-04  6.24E-04 |
| BAD | BAD, PIM2, BCL2L1, BCL2A1, YWHAH, YWHAZ, YWHAB, BCL2L2, S100A10, RPS6KA3, YWHAG, MAP2K5, YWHAQ, BCL2, SNCA, PRKACA, RPS6KA1, RPS6KA2, HRK, WASF1, YWHAE, BCL2L10, PAK7, MCL1, PPP1CA, PPP3CA, RPS6KA5, EWSR1, SFN, RAF1, MAPK8, PAK1, PIM1, PIM3, AKT1 | Signalling by NGF  NGF signalling via TRKA from the plasma membrane  Intrinsic Pathway for Apoptosis | 7.87E-13  6.87E-12  5.08E-10 | Neurotrophin signaling pathway  Oocyte meiosis  **MAPK signaling pathway**  Epstein-Barr virus infection | 1.86E-20  2.53E-14  1.33E-08  1.27E-08 |
| BMP7 | BMP7, ACVR1, ACVR2A, ENG, BMPR2, BMPR1A, NCOA3, ACVR2B, NOG, BMPR1B, GDF7, CHRDL2, SOSTDC1 | Signaling by BMP  Signaling by NODAL  Regulation of Signaling by NODAL | 1.02E-14  1.58E-04  3.72E-05 | TGF-beta signaling pathway | 9.54E-18 |
| CAPN3 | LNC, CAPN3, DYSF, NECAB2, YWHAQ, TTN |  |  |  |  |
| CCL18 | CCL18, UNC119, C14orf1, EEF1A1, TP53, TLE1, CRMP1 |  |  |  |  |
| **CCL2** | DARC, CCL2, VCAN, MMP1, MMP8, MMP3, CCR10, CCR1, CCR2, CCR5, CCRL1, CCBP2 | 1.Peptide ligand-binding receptors  2.Chemokine receptors bind chemokines  3.Degradation of the extracellular matrix  4.Activation of Matrix Metalloproteinases | 2.89E-10  3.13E-12  2.02E-05  2.02E-05 |  |  |
| CCL8 | CCL8, CCR1, CCR2, CCR3, CCR5, VCAN, MMP3, CCRL1, CCBP2, DARC | Chemokine receptors bind chemokines | 4.04E-11 |  |  |
| **CCR1** | CCL3, CCL4, CCL5, CCR1, PLP2, CREB3, JAK1, STAT1, STAT3, CCL26, CCL2, CCL7, CCL14, CCL15, CCL16, CCL3L1, CCL8, CCL23, GNA14, TPST1, TPST2 | 1.Peptide ligand-binding receptors  2.**Chemokine receptors bind chemokines**  3.Regulation of IFNG signaling  4.Interleukin-6 signaling | 2.89E-10  1.61E-14  4.37E-04  1.85E-06 | **Cytokine-cytokine receptor interaction**  **Chemokine signaling pathway** | 7.97E-15  7.40E-21 |
| CD27 | CD27,TRAF2,TRAF3,TRAF5,SIVA1,CD70 |  |  |  |  |
| CD36 | YES1, LYN, ITGB3, COL1A1, COL1A2, ITGB1, FYN, CD9, ITGA6, THBS1, SRC, MATK, ITGA2B | 1.Platelet activation, signaling and aggregation  2.Cell surface interactions at the vascular wall  3.Integrin cell surface interactions | 3.67E-11  3.03E-14  8.86E-13 | Focal adhesion  ECM-receptor interaction | 1.95E-11  1.10E-10 |
| **CD3E** | CD3E,CD3EAP,PIK3R1,ZAP70,CD3D,TRB@,SYK,SHC1,NCK2,TOP2B,CD79B,NCL,UNC119,NCK1,TRAT1,CD3G,PTPN22,LCK | 1.TCR signaling  2.Downstream TCR signaling  3.Generation of second messenger molecules | 2.14E-14  6.89E-11  7.26E-12 | 1.ErbB signaling pathway  2.T cell receptor signaling pathway  3.Pathogenic Escherichia coli infection  4.Primary immunodeficiency | 1.62E-05  2.95E-12  1.36E-04  5.05E-07 |
| CD5 | CD5,CD72,RASA1,CSNK2A1,CD79A,CD79B,FYN,LCK,CBL,PIK3R1,PTPN6,ZAP70,PRKCA,PRKCB,PRKCG,CD6,CD247,CD4,CD2,DYNLT3,HNRNPU,CAMK2D | 1.Costimulation by the CD28 family  2.Signaling by SCF-KIT  3.Signaling by the B Cell Receptor (BCR) | 4.11E-08  1.05E-07  1.62E-06 | 1.**Natural killer cell mediated cytotoxicity**  2.T cell receptor signaling pathway  3.ErbB signaling pathway | 2.49E-10  1.06E-09  2.87E-07 |
| CIT | RHOC,RHOA,GRIN2D,DLG4,DISC1,GRIN1,CIT | 1.Sema4D in semaphorin signaling  2.Ras activation uopn Ca2+ infux through NMDA receptor  3.CREB phosphorylation through the activation of CaMKII | 2.75E-04  2.25E-07  1.58E-07 | Cocaine addiction  Nicotine addiction | 1.94E-06  2.39E-04 |
| CNOT8 | CNOT8,BTG2,CNOT3,CNOT4,CNOT1,CNOT6,BTG1,CNOT2 | Deadenylation of mRNA | 4.91E-13 | RNA degradation | 1.91E-16 |
| COPE | COPA, COPG2, ARF1, COPB1, COPG, COPE |  |  |  |  |
| CUL4A | CUL4A,CAND1,DDB1,SKP2,CDKN1B,RBX1,CHEK1,DDB2 | 1.Formation of incision complex in GG-NER  2.Cyclin D associated events in G1  3.Global Genomic NER (GG-NER) | 1.86E-04  5.98E-04  4.61E-04 | Nucleotide excision repair | 8.98E-08 |
| **CYBA** | CYBA,NCF4,NCF2,RAC1,NOX1,NCF1,CYBB | 1.Antigen processing-Cross presentation  2.Cross-presentation of particulate exogenous antigens (phagosomes)  3.Latent infection of Homo sapiens with Mycobacterium tuberculosis  4.Phagosomal maturation (early endosomal stage) | 1.04E-07  5.13E-12  3.39E-09  3.39E-09 | 1.**Phagosome**  2.Osteoclast differentiation  3.Leukocyte transendothelial migration  4.Leishmaniasis | 4.94E-12  1.32E-12  6.50E-13  6.26E-07 |
| DNAJA3 | DNAJA3,SMAD9,RAD51,HSPA8,ZBTB22,RASA1,HSPA1A,IFNGR2,JAK2,MET,RNF34,ATXN1,FAM131C,NTRK1,USP7,COIL,GFI1B | Regulation of IFNG signaling | 2.39E-04 |  |  |
| DTNA | DTNA,GFI1B,DRP2,DMD,SNTB1,SNTB2,SNTA1,UTRN,ACTA1,DTNBP1,SYNM,KCNJ12,SNTG1,SNTG2,SYNC |  |  |  |  |
| **DUSP1** | DUSP1,MAPK14,HSPA4,SKP2,CKS1B,MAPK1,UBB,MAPK8,MAPK3,MAPK12 | 1.Toll-like receptor signaling pathway  2.Fc epsilon RI signaling pathway  3.Shigellosis  4.Salmonella infection  5.Epithelial cell signaling in Helicobacter pylori infection | 2.52E-07  7.88E-08  1.85E-08  1.07E-07  1.42E-04 | 1.Toll Like Receptor 3 (TLR3) Cascade  2.**MyD88:Mal cascade initiated on plasma membrane** | 8.30E-07  1.16E-06 |
| **FCGR2B** | FCGR2B,PTPN6,INPPL1,LY6E,CRP,C14orf1,INPP5D,APCS,LYN,BLK,MAPK1,MAPK3 | 1.Signaling by Interleukins  2.Signaling by SCF-KIT  3.Growth hormone receptor signaling | 2.11E-07  3.39E-06  1.94E-08 | 1.B cell receptor signaling pathway  2.Fc gamma R-mediated phagocytosis | 3.68E-14  8.46E-11 |
| FGFBP1 | FGFBP1,FGF1,FGF2,UBQLN4,HSPG2,FIBP | 1.FGFR2 ligand binding and activation  2.SHC-mediated cascade | 1.78E-05  5.59E-05 |  |  |
| **FOS** | BCL3, CSNK2A1, CSNK2A2, JUNB, STAT1, SMAD3, ATF2, LMNA, DDIT3, CEBPG, HNF1A, RUNX1, MITF, NFKB1, RELA, ETS1, FOS, SUMO1, SUMO2, SUMO3, SUMO4, ATF7, EEF1D, RB1, SMARCD1, MAP3K7, NCOA1, NFATC3, TSC22D3, RPS6KA4, GATA4, BATF, COBRA1, USF2, NCOR2, EPHB2, PRKACA, PSMC5, RPS6KA1, GTF2F2, XBP1, ELK1, TAF1, TBP, RUNX2, ELK4, USF2, NCOR2, EPHB2, PRKACA, PSMC5, RPS6KA1, SMARCD1, MAP3K7, NCOA1, NFATC3, TSC22D3, RPS6KA4, GATA4, BATF, COBRA1 | 1.Toll Like Receptor 3 (TLR3) Cascade  2.**MyD88:Mal cascade initiated on plasma membrane**  3.MAP kinase activation in TLR cascade  4.RNA Polymerase II Transcription Initiation And Promoter Clearance | 8.58E-08  1.53E-07  3.94E-06  9.86E-04 | 1.HTLV-I infection  2.**MAPK signaling pathway**  3.Epstein-Barr virus infection | 1.46E-09  2.41E-07  1.37E-08 |
| GCM1 | GCM1,HDAC5,HDAC1,HDAC4,HDAC3,CREBBP | Signaling by NOTCH | 1.55E-09 | Notch signaling pathway | 5.50E-04 |
| GLS2 | GLS2,SNTA1,PAG1,DLG1,DLG2,DLG3,INADL,RGS3,CASK,TAX1BP3,DLG4 | 1.Activation of Kainate Receptors upon glutamate binding  2.NrCAM interactions | 4.81E-06  2.85E-08 |  |  |
| HIST2H2BE | HIST2H2BE,RCC1,PTMA,TGM2,TBL1X,HIRA,KPNA1,HSPD1,GADD45A,LALBA,TNPO1,HIRIP3,SAP30,DYRK2,NAP1L4,BRD7,TBL1XR1,AKT1,ATF2 | 1.Circadian Clock  2.NOTCH1 Intracellular Domain Regulates Transcription | 4.95E-05  0.002 |  |  |
| HOXC8 | HOXC8,JUN,SMAD4,BMPR1A,SMAD1,BTG2,SMAD6,GMNN,HOMEZ | Signaling by BMP | 2.33E-09 | TGF-beta signaling pathway | 4.53E-07 |
| IL2RA | STAT3,IL2RB,IL2,IL2RA,ICAM1,NFKB1,CD4,STAT5B | 1.Downstream TCR signaling  2.Signaling by Interleukins | 0.001  8.33E-10 | Measles  Acute myeloid leukemia | 2.63E-09  3.94E-05 |
| INADL | KCNJ15, KCNJ10, GRIN2D, SCN4A, CACNG2, PARD3, GRIN2B, GRIN2A, GRIN2C, CLDN1, NLGN2, MPP5, ACCN3, NRXN2, CRB3, TJP3, CRIPT, KCNA4, CNKSR2, KIF1B, MAPK12, SCN5A, GLS2, KCNJ2, PAX6, PRDM16, DDX18 | 1.Transmission across Chemical Synapses  2.Cell-cell junction organization  3.Ras activation uopn Ca2+ infux through NMDA receptor | 7.98E-10  1.73E-05  1.69E-07 | 1.Amyotrophic lateral sclerosis (ALS)  2.Long-term potentiation  3.Cocaine addiction | 2.90E-07  3.98E-05  1.04E-05 |
| INHBB | INHBB,ACVR1,ACVR2A,INHBA,INHA,INHBB,IGSF1,INHBC,ACVR1B,ACVR2B,ACVR1C | 1.Glycoprotein hormones  2.Peptide hormone biosynthesis  3.Signaling by NODAL | 5.06E-10  1.02E-09  3.12E-09 | TGF-beta signaling pathway | 4.99E-13 |
| **IRS2** | IRS2,PIK3R1,PLCG1,PTPN11,TYK2,ATP2A1,BCL2L1,JAK3,SHC1,UBTF,PTPN6,PIK3CD,PIK3R2,SOCS1,PIK3R3,SOCS7,NTRK1,EPOR,IGF1R,IL4R,JAK1,PTPRF,GRB2,YWHAZ,YWHAE,SOCS6,YWHAG,SOCS3,JAK2,ATP2A2,MPL,CRK,PIK3CA,FES,YWHAB,INSR | 1.Cytokine Signaling in Immune system  2.Signaling by Interleukins  3.Interleukin-3, 5 and GM-CSF signaling | 3.97E-20  1.50E-22  4.15E-23 | 1.Jak-STAT signaling pathway  2.Neurotrophin signaling pathway  3.Insulin signaling pathway  4.Epstein-Barr virus infection | 1.48E-22  5.53E-19  1.10E-13  1.63E-11 |
| **JUN** | JUN, BCL3, BCL6, BRCA1, STAT3, TOP1, MAPK11, ELF3, NCOA2, ATF1, ATF2, MAPK10, DDIT3, TOP2A, EGR1, TCF4, SNAPC5, ESR1, FOSL1, NR3C1, NFE2L2, HOXA9, HOXC8, RUNX1, MYOD1, HNRNPM, NFE2L1, RELA, ETS1, ETS2, SKI, FOS, SPI1, POU1F1, MAF, RB1, NR5A1, NFYA, SP1, GTF2F2, ABL1, TPM1, HCFC1, TAF1, AR, TBP, CREBBP, RUNX2, STAT1, STAT4, HMGA1, PRKDC, SMAD4 PIN1, MAPK8, NACA, SMAD2,TDG, FOSL2, SMARCD1, VDR, MAPK3, SUMO1, NCOA3, NRIP1, TGIF1, NCOA1, EP300, MAPK9, TSC22D3, TCF20, SMAD3, DHX9, ATF3, HIF1A, TRIP4, MYBBP1A, PRKD1, DDX21, SPIB, PACS1, ATF4, HHEX, COPS5, MAFB, RFWD2, EN1, GTF2B, HDAC3, UBE2I, RBM39, RAGE, MAPKAPK5, BATF, CREB5, COBRA1, TPM2, PIAS1, RPL18A, JDP2, SNRK, SUMO2, SUMO3, FBXW7, CSNK2A1, ERG, SOX8,SOX10, SUMO4, ITCH, SMARCD3, NTRK3, IRAK1, ELOF1, SNAPC5, GATA2,UBB | 1.Generic Transcription Pathway  2.Innate Immune System  3.Toll Like Receptor 3 (TLR3) Cascade  4.**MyD88:Mal cascade initiated on plasma membrane** | 3.86E-07  6.29E-08  3.87E-11  1.06E-10 | 1.HTLV-I infection  2.Epstein-Barr virus infection  3.**MAPK signaling pathway**  4. Osteoclast differentiation  5.Epithelial cell signaling in Helicobacter pylori infection | 9.07E-14  9.24E-10  5.54E-05  2.15E-08  9.88E-07 |
| JUNB | JUNB,BCL6,BRCA1,JUNB,NINL,FOSL1,NFE2L1,FOSB,MAPK14,SMAD4,MAPK8,FOSL2,SMAD3,BATF,JDP2,FOS,ESR1 | 1.Signaling by TGF-beta Receptor Complex  2.MAP kinase activation in TLR cascade  3.Activation of the AP-1 family of transcription factors | 9.79E-05  3.94E-05  2.34E-07 | Osteoclast differentiation  Pertussis  Colorectal cancer  Pancreatic cancer | 9.57E-10  3.28E-04  4.16E-06  2.78E-04 |
| KCNK15 | KCNK15,YWHAG,YWHAH,YWHAE,YWHAB,YWHAZ,YWHAQ,SFN | Rap1 signalling  Signaling by Hippo | 1.00E-04  1.70E-04 | 1.Cell cycle  2.Oocyte meiosis  3.Neurotrophin signaling pathway | 1.05E-12  2.04E-10  4.40E-10 |
| KCNK3 | KCNK3,COPB1,YWHAB,S100A10,YWHAZ,YWHAQ,YWHAE,YWHAH,YWHAG,SFN | Rap1 signalling  Signaling by Hippo | 4.02E-05  6.82E-05 | 1.Cell cycle  2.Oocyte meiosis  3.Neurotrophin signaling pathway | 1.05E-12  2.04E-10  4.40E-10 |
| KRT19 | KRT19,TUBG1,KRT15,KRT6B,PCM1,FANCG,HGS,ABI2,FAM107A,EXOC8,SFI1,ZNF638,C10orf10,DGCR6L,USHBP1,DMD,PNN | NA |  | NA |  |
| LIMK2 | LIMK2,LIMK1,CFL1,GSN,ROCK1,CDC42BPA,PARD3,HIPK3 | 1.Apoptotic cleavage of cellular proteins  2.Semaphorin interactions | 6.58E-04  2.23E-07 | Fc gamma R-mediated phagocytosis | 8.11E-07 |
| MTA2 | MTA2,SATB1,RBBP7,RBBP4,MBD3,TP53,SPEN,APPL1,APPL2 | NA |  | NA |  |
| MKNK1 |  |  |  |  |  |
| MUC7 | SELL,MUC7,LTF,GALNT10,GALNT14,GALNT12,HTN3,AMY1A | O-linked glycosylation of mucins | 3.40E-07 | Mucin type O-Glycan biosynthesis | 2.21E-06 |
| **NCF4** | TXN,NCF2,MSN,CORO1A,XRCC6,PRKCD,CYBA,NCF1,CYBB | 1.Cross-presentation of particulate exogenous antigens (phagosomes)  2.Latent infection of Homo sapiens with Mycobacterium tuberculosis  3.Phagosomal maturation (early endosomal stage) | 4.56E-08  4.81E-06  4.81E-06 | Leukocyte transendothelial migration  Leishmaniasis | 1.10E-07  8.65E-05 |
| NEK9 | RAN,NEK6,NEK7,BICD2,NEK9,SSRP1,SUPT16H,NEK9,MBP | Pausing and recovery of Tat-mediated HIV-1 elongation | 7.33E-05 |  |  |
| **NOTCH3** | NOTCH3,PSEN2,MAML2,MAML3,CHUK,RBPJ,JAG1,JAG2,SNW1,MAML1,DLL1,KAT2B,PSEN1 | 1.Signaling by NOTCH  2.Regulated proteolysis of p75NTR  3.NRIF signals cell death from the nucleus | 6.24E-21  2.10E-04  4.55E-04 | Notch signaling pathway | 9.62E-26 |
| PGF | NRP2, FLT1, NRP1, PGF, VEGFA | VEGF ligand-receptor interactions | 3.08E-12 |  |  |
| PLEKHB1 | SMAD1,ACVR1,BMPR1B,GNGT1,PLEKHB1,TGFBR1 | Signaling by BMP | 8.15E-05 |  |  |
| PNKP | PNKP,XRCC1,KRTAP4-12,XRCC4,SMAD5,MAGEA11,TRIM37,MYOZ1 | NA | NA | NA | NA |
| PPP2R3A | PPP5C,ATXN7L2,RBL1,PPP2R3A,HMGB1,AKAP9,CDC6 | G0 and Early G1 | 1.04E-04 |  |  |
| **PRKCA** | KIT, OPRD1, RHOA, SPP1, PAM, PFKFB2, KCNE1, PTPN11, PRKCA, RRAD, RHO, DDX5, SRC, TP53, VCL, VTN, NCF1, GJB1, RPL10, ITGB2, PTPN12, SNAP25, NFATC1, DLX3, CASR, GPM6A, DGKZ, ADD3, GMFB, PA2G4, NRGN, DNM1, PLD1, HAND1, CFTR, CYTH2, RGS7, SNAP23, OCLN, STXBP1, SPAG1, PEA15, GRM1, RGS19, CD163, TRPV6, GSK3A, PLCB1, HSPB8, PEBP1, CHAT, MARCKS, PRKG1, PTGIR, EDF1, ADAP1, TNNI3, HABP4, GABRR1, TRPC3, PPP1R14A, THOC5, GRIA2, HMGB1, RARA, SDC4, TNNT2, ATP2B1, ADRBK1, PTPN6, SLC6A9, KCNQ2, GRM5, F11R, CDC42, ATP2B2, CBL, SHC1, DGKD, ACTA1, GJA1, DVL2, CREM, CORO1B, EZR, TOP2A, FLNC, RALBP1, EEF1D, EGFR, EIF4E, EWSR1, SLC1A1, ITGB1, GABRG2, GFAP, GRIA4, GRIA1, GRIN2B, GRIN2A, GFPT1, GNA15, HES1, HSPA1A, SDC2, HLA-A, CD9, ADRA1B, ITPKA, ITPKB, INSR, KRT18, LMNA, LMNB1, BCL2, ANXA2, CD5, LCK, MGMT, MBP, MYOD1, NOS1, APLP2, HMGN2, HMGN1, RAF1, GNB2L1, PRKCZ, SCTR, SEMG1, SEMG2, TERT, XK, SYK, TIAM1, AVPR1A, RGS2, MYLK, C1QBP, YWHAZ, BTG2, TEP1, ARHGEF1, OGG1, RAC1, PLD2, HAND2, CISH, FSCN1, DLG4, GNA12, AKAP5, AKAP12, YWHAG, POLB, ADCY5, SDPR, KCNE4, AFAP1, TRIM29, PDLIM7, STXBP3, GABRR2, BTK, ANXA7, FAS, ITGB4, PICK1, PPARA, FLNA, NUMB, HSP90AA1, ENTPD5 | 1.Hemostasis  2.Platelet activation, signaling and aggregation  3.Transmission across Chemical Synapses  4.Gastrin-CREB signalling pathway via PKC and MAPK | 3.08E-11  3.07E-10  1.98E-09  3.33E-05 | 1.Focal adhesion  2.Calcium signaling pathway  3.Glutamatergic synapse  4.Regulation of actin cytoskeleton  5.**Chemokine signaling pathway**  **6.Endocytosis**  **7.Bacterial invasion of epithelial cells**  8.**Natural killer cell mediated cytotoxicity** | 1.68E-07  1.15E-06  1.05E-08  9.45E-06  5.22E-05  1.04E-04  5.72E-06  1.07E-05 |
| PRMT2 | RXRA,NCOA6,PGR,THRB,DMRTB1,CPSF7,PRMT2,RB1,ESR1,BAT2,HNRNPUL1,ESR2,NCOA1 | 1.Nuclear Receptor transcription pathway  2.BMAL1:CLOCK/NPAS2 Activates Circadian Expression | 8.09E-09  2.11E-05 |  |  |
| PVRL3 | PVR, PARD3, PVRL1, PVRL3, PVRL2, MLLT4 | Nectin/Necl trans heterodimerization  Cell-cell junction organization | 7.70E-12  4.41E-13 | Adherens junction | 1.15E-09 |
| **PYCARD** | PYCARD,MEFV,SRF,NLRP3,NLRC4,CASP1,NLRP12,PRKAR1A,NLRP1,PYDC2,POP1 | 1.Inflammasomes  2.Nucleotide-binding domain, leucine rich repeat containing receptor (NLR) signaling pathways | 2.07E-15  1.37E-12 | NOD-like receptor signaling pathway | 4.12E-11 |
| RALB | RALB,CALM1,REPS2,RALBP1,REPS1,RGL4,PLCD1 | NA | NA | NA | NA |
| RALBP1 | RALBP1,RALA,RALB,REPS2,AP2M1,RAC1,HOOK2,REPS1,CCNB1,SYNJ2BP,IKBKG,CASP8,TRAF2,FADD,PRKCA | 1.Activation of Pro-Caspase 8  2.Extrinsic Pathway for Apoptosis  3.TNF signaling  4.Caspase-8 is formed from procaspase-8  5.Death Receptor Signalling | 3.21E-07  1.09E-06  1.34E-07  3.21E-07  1.09E-06 | Pancreatic cancer | 6.81E-08 |
| RANBP2 | RANBP2,OPN1MW,OPN1LW,RAN,HDAC4,MDM2,IPO5,RANGAP1,XPO1,KPNB1,TNPO1,XPOT,NUP62,UBE2I,PARK2 | 1.Interactions of Rev with host cellular proteins  2.Rev-mediated nuclear export of HIV-1 RNA  3.NEP/NS2 Interacts with the Cellular Export Machinery  4.Export of Viral Ribonucleoproteins from Nucleus | 6.17E-11  5.31E-09  3.38E-07  3.90E-07 |  |  |
| RARG | RARG,SMAD3,ITGB1BP2,MAP6,NR0B2,PNRC1,PNRC2,RXRB,RXRA,RXRG,NCOR2,NCOA3,NCOA1,HMGA1 | Nuclear Receptor transcription pathway  BMAL1:CLOCK/NPAS2 Activates Circadian Expression | 8.09E-09  2.11E-05 | Thyroid cancer | 1.80E-06 |
| RASGRP2 | RASGRP2,KRAS,FAM118B,PTCHD2,NRAS,RAP1A | 1.ARMS-mediated activation  2.Prolonged ERK activation events  3.Frs2-mediated activation  4.SOS-mediated signalling | 6.33E-08  9.02E-08  7.60E-08  2.69E-05 | Long-term potentiation  Renal cell carcinoma  Thyroid cancer  **Bladder cancer** | 5.40E-06  5.40E-06  1.25E-04  2.64E-04 |
| RHOB | RHOB,PGGT1B,PDE6D,ARHGDIG,RHPN2,ARHGEF3,PPP2CA,FNTA | NA | NA | NA | NA |
| RND2 | RND2,RACGAP1,UBXN11,FNBP1,VPS4A,MEOX2 | NA | NA | NA | NA |
| RRN3 | RRN3,TAF1B,TAF1C,POLR1B,POLR1A,EIF3L,MYO1C,TAF1B,TAF1C,POLR1B,POLR1A,EIF3L,MYO1C |  |  |  |  |
| S100P | AGER,S100P,EZR,S100A4,S100A2,CACYBP,S100Z,SUGT1,S100A1 | NA | NA | NA | NA |
| SCAP | SREBF2,SCAP,INSIG1,INSIG2,PGRMC1 | NA | NA | NA | NA |
| SERPINB6 | SERPINB6,PLG,PROC,F2,PLAU,F10,KLK2 | 1.Removal of aminoterminal propeptides from gamma-carboxylated proteins  2.Formation of Fibrin Clot (Clotting Cascade)  3.Intrinsic Pathway | 5.58E-08  2.29E-06  1.00E-04 | Complement and coagulation cascades | 2.98E-09 |
| SERPINC1 | SERPINC1,SDC2,KLK2,PLG,F2,KLK6 | 1.Common Pathway  2.Formation of Fibrin Clot (Clotting Cascade)  3.Regulation of Insulin-like Growth Factor (IGF) Activity by Insulin-like Growth Factor Binding Proteins (IGFBPs) | 4.48E-05  2.43E-04  6.69E-05 |  |  |
| SFN | SFN,ZFP36,TSC2,BAX,TRIM25,CDK1,MARK3,BAD,CHST1,WDYHV1,TBL3,PLK4,GPRIN2,PLEKHF2,FAM189A2,KIAA0408,MAP3K5,HNRNPD,EIF2S1,EIF4B,EEF1A1,NR3C1,BCR,HDAC5,CCAR1,MST1R,YWHAG,CDC25B,ING1,KCNK3,KCNK9,KCNK15,EGFR,ABL1,MDM4,RFFL | 1.GAB1 signalosome  2.PI3K events in ERBB2 signaling  3.PKB-mediated events  4.Intrinsic Pathway for Apoptosis | 4.05E-04  5.28E-04  0.004  0.005 | 1.p53 signaling pathway  2.Amyotrophic lateral sclerosis (ALS) | 2.38E-06  6.95E-04 |
| SKP2 | SKP2,UBB,WEE1,E2F1,CDKN1B,CDKN1C,MYBL2,SKP1,CKS1B,CDC34,CDK2,CCNA2,CCNE1,RB1,TCF3,ORC1L,CCNT1,CUL1,PFDN1,CDT1,GPS1,CDK9,FZR1,CUL4A,DDB1,MYC,MYB,NLK,DUSP1,TAL1,ELF4 | 1.Mitotic G1-G1/S phases  2.S Phase  3.G1/S Transition | 3.79E-20  1.11E-19  1.44E-19 | 1.Cell cycle  2.Small cell lung cancer  3.Ubiquitin mediated proteolysis | 5.77E-18  1.33E-09  9.93E-07 |
| SLC9A3R2 | SLC9A3R2,PTH1R,RDX,SLC9A3,ATP2B2,TAZ,PLCB3,ADRB2,P2RY1,PRKACA,SIAH1,CFTR,PODXL,SGK1,ACTN4,PDPK1,EZR,SLC22A4,SLC22A5,SLC22A9,SLCO1A2,LPAR1,SGK3,SRY,SLC34A1,KCNJ1,WWTR1,ADORA2B,TBC1D10A,SLCO3A1,PTEN,LPAR2 | 1.PI3K/AKT activation  2.Nucleotide-like (purinergic) receptors  3.Lysosphingolipid and LPA receptors | 0.010  0.0023  4.09E-04 | 1.Gastric acid secretion  2.Aldosterone-regulated sodium reabsorption  3.Bile secretion  4.Endocrine and other factor-regulated calcium reabsorption | 4.65E-06  1.09E-05  9.72E-05  6.35E-04 |
| SMG5 | SMG5,TERT,UPF1,SMG1,SMG6,SMG7 |  |  | mRNA surveillance pathway | 3.35E-09 |
| SNAPC5 |  | RNA Polymerase III Transcription Initiation | 7.81E-05 |  |  |
| SOCS7 | SOCS7,PLCG1,GRB2,NCK1,SORBS3,EGFR,PIK3R1,IRS2,TUBA1A,TUBB2C,PIK3R2,IRS4 | 1.PI3K Cascade  2.TCR signaling  3.Signaling by constitutively active EGFR  4.Antigen Activates B Cell Receptor Leading to Generation of Second Messengers | 4.37E-06  1.54E-06  1.18E-08  1.08E-07 | 1.ErbB signaling pathway  **2.Glioma**  3.Aldosterone-regulated sodium reabsorption | 2.73E-09  4.67E-08  5.16E-07 |
| TCEB3B | TCEB3B,TCEB2,TCEB1,ZNF165,TRAF2,DVL2,CBX5,TRIM37,CALCOCO2,SOHLH2,ZBTB43,KRTAP4-12,C19orf50,CEP70,COIL | 1.Pausing and recovery of HIV-1 elongation  2.HIV-1 elongation arrest and recovery  3.Tat-mediated HIV-1 elongation arrest and recovery  4.Regulation of Hypoxia-inducible Factor (HIF) by Oxygen  5.Cellular responses to stress | 5.39E-04  5.39E-04  5.07E-04  3.60E-04  3.60E-04 |  |  |
| TFF1 | TFF1,GKN2,UBQLN1,UBQLN4,MUC6 | NA | NA | NA | NA |
| TNPO1 | SRP19,NUP98,RAN,RANBP2,HNRNPD,HIST2H2BE,PABPN1,NXF1,RGPD5,HIST3H3,TNPO1,NUP214,HNRNPA1,RPL5,RPS7,NUP153,RPL23A,ELAVL1 | 1.Processing of Capped Intron-Containing Pre-mRNA  2.Transport of Mature mRNAs Derived from Intronless Transcripts | 1.30E-09  2.56E-08 |  |  |
| UBE2B | UBE2B,CRMP1,UNC119,UBR1,MED31,RAD18,UBR2,CNOT4 | NA | NA | NA | NA |
| UBQLN4 | UBQLN4,SERPINH1,ATXN1,DTX2,C1QTNF1,ERP27,ZG16B,SMCR7,ITPRIPL1,CCDC107,ANKRD13D,NOTCH2NL,NOMO3,UBQLN4,PMEPA1,PELI2,PBXIP1,MIF4GD,KLHDC5,SCAF1,RIC8A,MOAP1,CCDC136,CCDC14,RSRC2,C11orf49,FA2H,CCDC134,CCDC33,GKAP1,ADAM33,ADPGK,USMG5,C1orf94,ATPIF1,SMAD9,EDN1,ELF5,FKBP2,GABRD,GDI1,GPX7,HK2,IGFBP6,IGHM,IGLC1,IMPDH2,STMN1,COL8A1,COPB1,MDK,CD99,MLLT6,NME3,NPHP1,NPPA,OAT,SERPINE1,PCDH8,SERPINI2,PIN1,PPIB,PPIC,SRGN,QSOX1,PTPRN,PTPRN2,RAD23A,ROBO2,RPN1,CSTF2,DMPK,EEF1A1,PTN,RXRA,UNC119,CCL21,SEMG1,SFRS2,SCG5,SPINT1,SPP1,HSPA13,TFF1,TGFB1I1,TRAF2,UROS,VIP,LAT2,ZNF205,SCG2,AREG,BAT3,SMARCB1,NAE1,ZBTB22,MDM2,RBM10,FZD7,PIP4K2B,CACNA1G,HGS,PNMA1,PDLIM7,CRIPT,PICK1,DAZAP2,FGFBP1,ARL4C,STAM2,NXF1,AGR2,RAI2,RNPS1,PDIA5,ERP29,YWHAQ,IMMT,CPSF6,PLA2G16,TRIM32,SCMH1,TNRC6B,CSTF2T,ZFPM2,NOMO1,SPAG8,HAVCR1,DKK3,PCDH17,TRIB2,UBQLN2,UBQLN1,EFEMP2,CEND1,PLUNC,ZDHHC3,WAC,CYB5R1,EPDR1,UBR7,KLHL26,FAM48A,PRPF40A,EAPP,C19orf10,RNF11,RUNX1T1,CDSN | 1.Cleavage of Growing Transcript in the Termination Region  2.RNA Polymerase II Transcription Termination | 0.004  0.004 | mRNA surveillance pathway | 6.52E-04 |
| USP4 | USP4,RB1,TRIM21,NOLC1,RBL1,TP53BP2 |  |  |  |  |
| ZFP36 | ZFP36,YWHAH,NUP214,CCDC85B,HMGB1,MAPK1,YWHAB,SFN,YWHAG,EXOSC8,UPF2,MAPKAPK2,EXOSC6,XRN1,EDC3 | 1.Regulation of mRNA Stability by Proteins that Bind AU-rich Elements  2.Destabilization of mRNA by Tristetraprolin (TTP)  3.Destabilization of mRNA by Butyrate Response Factor 1 (BRF1) | 5.70E-11  1.23E-13  5.59E-11 | RNA degradation | 1.43E-05 |
