# Supplementary material for: Dynamic Modularity of Host Protein Interaction Networks in Salmonella Typhi Infection
Source: PLoS One. 2014 Aug 21;9(8):e104911. doi: 10.1371/journal.pone.0104911 (PMC4140748; doi:10.1371/journal.pone.0104911)
Supplement: Table S5 — List of enriched pathway term (analysed using REACTOME databases) that showed higher number of hits when searched in PubMed with the subject “pathways term and Salmonella / Salmonella Typhi.” (DOCX) [file pone.0104911.s008.docx]

Table S5: List of enriched pathway term (analysed using REACTOME databases) that showed higher number of hits when searched in PubMed with the subject “pathways term and Salmonella/Salmonella typhi”

| Sl no | Pathways term | PubMed Hits | | Frequency of occurrence in the dataset | Hub name |
| --- | --- | --- | --- | --- | --- |
|  |  | Salmonella | Salmonella Typhi |  |  |
| 1 | Cytokine Signaling in Immune system | 236 | 15 | 1 | IRS2 |
| 2 | Downstream TCR signaling | 125 | 7 | 2 | CD3E, IL2RA |
| 3 | Hemostasis | 113 | 19 | 1 | PRKCA |
| 4 | Cross-presentation of particulate exogenous antigens (phagosomes) | 82 | 10 | 2 | CYBA, NCF4 |
| 5 | Chemokine receptors bind chemokines | 67 | 5 | 3 | CCL2, CCL8, CCR1 |
| 6 | AKT activation | 24 | 1 | 2 | ATP2A2, SLC9A3R2 |
| 7 | Nuclear Receptor transcription pathway | 12 |  | 2 | PRMT2, RARG |
| 8 | Activation of Pro-Caspase 8 | 11 | 2 | 1 | RALBP1 |
| 9 | GTPases Rho GTPase cycle | 6 |  | 1 | ARHGDIG |
| 10 | Peptide ligand-binding receptors | 6 |  | 2 | CCL2, CCR1 |
| 11 | Intrinsic Pathway for Apoptosis | 4 |  | 2 | SFN, BAD |
| 12 | ARMS-mediated activation | 4 | 2 | 1 | RASGRP2 |
| 13 | Cell-cell junction organization | 2 |  | 2 | INADL, PVRL3 |
| 14 | Regulation of IFNG signaling | 2 |  | 2 | CCR1, DNAJA3 |
| 15 | Signaling by BMP | 2 |  | 3 | HOXC8, BMP7, PLEKHB1 |
| 16 | Antigen processing-Cross presentation | 2 | 1 | 1 | CYBA |
| 17 | Apoptotic cleavage of cellular proteins | 2 |  | 1 | LIMK2 |
| 18 | O-linked glycosylation of mucins | 1 |  | 1 | MUC7 |
| 19 | Signaling by NOTCH | 1 |  | 2 | NOTCH3, GCM1 |
| 20 | Circadian Clock | 1 |  | 1 | HIST2H2BE |
