# Supplementary material for: Dynamic Modularity of Host Protein Interaction Networks in Salmonella Typhi Infection
Source: PLoS One. 2014 Aug 21;9(8):e104911. doi: 10.1371/journal.pone.0104911 (PMC4140748; doi:10.1371/journal.pone.0104911)
Supplement: Table S6 — List of enriched pathway term (analysed using KEGG databases) that showed higher number of hits when searched in PubMed with the subject “pathways term and Salmonella / Salmonella Typhi.” (DOCX) [file pone.0104911.s009.docx]

Table S6: List of enriched pathway term (analysed using KEGG databases) that showed higher number of hits when searched in PubMed with the subject “pathways term and Salmonella/Salmonella typhi”

| Sl no | Pathways term | PubMed Hits | | Frequency of occurrence in the dataset | Hub name |
| --- | --- | --- | --- | --- | --- |
|  |  | Salmonella | Salmonella Typhi |  |  |
| 1 | Bacterial invasion of epithelial cells | 2459 | 146 | 3 | ARHGDIG, ARPC5, PRKCA |
| 2 | Endocytosis | 1089 | 146 | 1 | PRKCA |
| 3 | Chemokine signaling pathway | 375 | 19 | 2 | CCR1, PRKCA |
| 4 | Cytokine-cytokine receptor interaction | 348 | 24 | 1 | CCR1 |
| 5 | Phagosome | 231 | 10 | 2 | CYBA, NCF4 |
| 6 | Bile secretion | 217 | 44 | 1 | SLC9A3R2 |
| 7 | Regulation of actin cytoskeleton | 148 | 5 | 1 | PRKCA |
| 8 | Natural killer cell mediated cytotoxicity | 125 | 10 | 1 | PRKCA |
| 9 | Bladder cancer | 116 | 5 | 1 | RASGRP2 |
| 10 | MyD88:Mal cascade initiated on plasma membrane | 97 | 2 | 3 | DUSP1, FOS, JUN |
| 11 | Primary immunodeficiency | 60 | 2 | 1 | CD3E |
| 12 | Gastric acid secretion | 50 | 8 | 1 | SLC9A3R2 |
| 13 | MAPK signaling pathway | 43 |  | 3 | BAD, FOS, JUN |
| 14 | Glioma | 28 | 1 | 1 | SOCS7 |
| 15 | Fc gamma R-mediated phagocytosis | 16 | 4 | 2 | FCGR2B, LIMK2 |
| 16 | T cell receptor signaling pathway | 14 | 1 | 2 | CD3E, CD5 |
| 17 | Focal adhesion | 13 |  | 2 | CD36, PRKCA |
| 18 | Toll Like Receptor 3 (TLR3) Cascade | 9 |  | 3 | DUSP1, FOS, |
| 19 | Insulin signaling pathway | 9 |  | 1 | IRS2 |
| 20 | Calcium signaling pathway | 8 |  | 1 | PRKCA |
| 21 | Aldosterone-regulated sodium reabsorption | 6 | 1 | 3 | ATP2A2, SLC9A3R2, SOCS7 |
| 22 | B cell receptor signaling pathway | 5 | 2 | 1 | FCGR2B |
| 23 | Jak-STAT signaling pathway | 5 |  |  |  |
| 24 | Leukocyte transendothelial migration | 5 |  | 1 | IRS2 |
| 25 | Adherens junction | 4 |  | 2 | ARHGDIG , PVRL3 |
| 26 | Osteoclast differentiation | 3 |  | 3 | CYBA, JUN, JUNB |
| 27 | Complement and coagulation cascades | 1 |  | 1 | SERPINB6 |
